# Supplementary material for: The Burden of Late Effects and Related Risk Factors in Adolescent and Young Adult Cancer Survivors: A Scoping Review
Source: Cancers (Basel). 2021 Sep 28;13(19):4870. doi: 10.3390/cancers13194870 (PMC8508204; doi:10.3390/cancers13194870)
Supplement: Supplementary file 1 [file cancers-13-04870-s001.zip › cancers-1365340-supplementary.pdf]

The burden of late effects and related risk factors in adolescent and young adult cancer survivors: A scoping review

Supplementary Material

Table S1. Examples of keywords used in PubMed literature searches. Keywords were appropriately combined using Boolean operators and searches were limited to the year 2010 to current.

cancer, malign*, neoplasm, carcinoma, leukemia, leukaemia, lymphoma, sarcoma, melanoma
adolescen*, young adult, adolescent and young adult, teen*, AYA
Second malignant neoplasms<br>second neoplasm*, subsequent neoplasm*, second malignant neoplasm*, new primary cancer*, second cancer*
Chronic conditions and hospitalizations<br>hospitaliz*, hospitalis*, hospital admission*, chronic condition*, chronic disease*, chronic health condition*, chronic illness*<br>anxiety, depress*, mental health, psychosocial health, psychiatric<br>heart disease*, cardiovascular disease*, CVD, obesity, diabetes, metabolic syndrome<br>toxicity
Mortality<br>death, mortality, surviv*

Table S2. Risk factors associated with second malignant neoplasms (SMNs) among adolescent and young adult cancer survivors by tumor group (n=23).

Reference	Country of origin	Purpose	Data source	Cancer type	Diagnosis period	Age at diagnosis (years)	Survival entry date	Number of AYA participants	Follow-up time	Results	Statistically significant risk factors	Non-significant risk factors
Mixed-cancer cohort												
Aben, 2012 [21]	Netherlands	To evaluate trends in cancer incidence, survival and risk of second primary cancers in AYAs.	The Netherlands Cancer Registry	Any primary malignancy except basal carcinomas of the skin	1989-2009	15-29	At diagnosis	23,161	Median 6.8 years	Males had a higher standardized incidence ratio than females (SIR=3.1, 95% CI 2.7-3.6 vs. SIR=2.0, 95% CI 1.8-2.3 respectively). The highest statistically significant SIR among males was for SMNs of the breast (SIR=16.7, 95%CI 1.9 – 60.2), but the number of observed cases were only 2, followed by SMN of bone and soft tissue (SIR=5.2, 95% CI 2.9-8.4) and the gastrointestinal tract (SIR=5.2, 95% CI 3.6-7.4). The highest statistically significant SIR among females was for SMN of the bone and tissue (SIR=6.3, 95% CI 3.5-10.3).	Male sex (SIR higher than for female sex)	No
Henderson, 2012 [22]	27 North American Centres	To determine the risk of gastrointestinal SMN in childhood cancer survivors.	The Childhood Cancer Survivor Study	First primary malignancy: leukemia, CNS malignancy, HL, NHL, neuroblastoma, soft-tissue sarcoma, kidney cancer or bone cancer. SMN: Sarcoma	1970-1986	15-20	5-years post-diagnosis	2,487	Median 22.8 years	No risk factors reported.	None presented	NA
Zhang, 2012 [18]	Canada	To assess long-term risks of overall and cause-specific mortality and SMN among survivors of	Childhood, Adolescent and Young Adult Cancer Survivors (CAYACS); British Columbia	Any primary malignancy	1970-1995	20-24	5-years post-diagnosis	1,248	21,711 person-years	More cases of SMN than expected were observed among people who, especially, received RT (SIR 5.7, 95% CI 4.0-7.9; AER 4.5 per 1,000 person-years at risk) or CT (SIR 6.3, 95% CI 4.1-9.4; AER 3.9 per 1,000 person-years), or did not receive surgery (SIR	Radiation (SIR higher than for those who did not receive it), Chemotherapy (SIR	Certain primary cancers (Melanoma, Carcinomas)

Reference	Country of origin	Purpose	Data source	Cancer type	Diagnosis period	Age at diagnosis (years)	Survival entry date	Number of AYA participants	Follow-up time	Results	Statistically significant risk factors	Non-significant risk factors
		young adult cancer compared to the risk in the general British Columbia population and evaluate the effects of demographic and clinical factors on risk.	Cancer Registry							5.0, 95% CI 3.0-7.8; AER 3.5 per 1,000 person-years).	higher than for those who did not receive it), No surgery (SIR higher than for those who did not receive it), 5-19 years follow-up time (SIR higher than for those followed 20+ years),	
Lee, 2016 [17]	United States	To describe the incidence and characteristics of SMN in AYA cancer survivors compared to those in younger and older cancer survivors.	SEER	First Primary: leukemia, lymphoma, germ cell tumors (Testicular, Ovarian) , melanoma, thyroid, breast, sarcomas (soft tissue or bone). SMNs: Any cancer with malignant behaviour excluding basal cell and cutaneous squamous cell carcinomas	1973-2011	15-39	5-years post-diagnosis	148,558	2,526,854 person-years	The 30-year cumulative incidence of SMN was higher among those who received radiation therapy compared to those who did not (17.6%, (95% CI:16.9%-18.3%) vs. 12.4%, (95% CI:12%-12.7%). The highest AER was observed in patients who were diagnosed with primary breast cancer (SIR 2.1, AER 54.4) or primary HL (SIR 2.8, AER 48.6).	Type of primary cancer, Radiation therapy (vs. no RT)	No

Reference	Country of origin	Purpose	Data source	Cancer type	Diagnosis period	Age at diagnosis (years)	Survival entry date	Number of AYA participants	Follow-up time	Results	Statistically significant risk factors	Non-significant risk factors
Teepen, 2017 [19]	Netherlands	To evaluate the long-term risk of SMN in a cohort of 5-year childhood cancer survivors with a focus on chemotherapy agents and risk of solid tumors.	The Dutch Childhood Cancer Oncology Group–Long-Term Effects After Childhood Cancer cohort	Any primary malignancy	1963-2001	15-17	5-years post-diagnosis	401	Median 20.7 years	No risk factors reported.	None presented	NA
Hayek, 2018 [23]	Israel	To estimate the risk of second primary neoplasm and long-term mortality among 5+ year childhood cancer survivors relative to their counterparts in the general population.	Israel National Cancer Registry	Any primary malignancy	1980-2007	15-19	5-years post-diagnosis	1,765	Age-specific follow-up NR	No risk factors reported.	None presented	NA
Bright, 2019 [24]	England	To investigate the risk of specific subsequent	Teenage and Young Adult Cancer Survivor Study	Breast, cervical, testicular, HL, NHL, melanoma, CNS (intracranial),	1971-2006	15-39	5-years post-diagnosis	197,827	Median 16.8 years	For women, statistically significant SIRs for the development of any subsequent primary neoplasm after specific cancers ranged from 1.3 (95% CI 1.1-1.6) for head and neck and 1.3	Younger age at diagnosis, Type of primary cancer,	No

Reference	Country of origin	Purpose	Data source	Cancer type	Diagnosis period	Age at diagnosis (years)	Survival entry date	Number of AYA participants	Follow-up time	Results	Statistically significant risk factors	Non-significant risk factors
		primary neoplasms after each of 16 types of AYA cancer.	(Teenage and Young Adult Cancer Survivor Study), United Kingdom	colorectal, thyroid, soft-tissue sarcoma, ovarian, bladder, other female genital cancers, leukemia, and head and neck						(95% CI 1.2-1.5) for CNS to 3.1 (95% CI 2.9-3.3) for HL. For men, significant SIRs ranged from 1.2 (95% CI for 1.1-1.4) for bladder to 2.6 (95% CI 2.4-2.8) for HL and 2.6 (95% CI 2.1-3.1) for leukemia. Excess risk of developing a subsequent primary neoplasm increased with follow-up time from diagnosis. For the development of any SMN after a specific first primary cancer diagnosis, SIRs ranged from 1.1 (95% 1.1-1.2) for breast cancer to 2.0 (95% CI 1.9-2.0) for lung cancer to 3.0 (95% CI 2.6-3.5) meninges.	Earlier decade of diagnosis, Time from diagnosis	
Chao, 2019 [25]	United States	To provide a comprehensive assessment of SMN risk in survivors of AYA cancer using study methods that minimized confounding to inform survivorship care planning for survivors of AYA cancer.	SEER	Any primary malignancy	1990-2012	15-39	2-years post-diagnosis	10,574	Median 7.7 years	Older age at diagnosis (30-39, IRR 1.79, 95% CI 1.21-2.65), female sex (IRR 1.31 95% CI 1.09-1.57), advanced stage at diagnosis (Stage II, IRR 1.35, 95% CI 1.11-1.65) , and exposure to RT (IRR 1.50, 95% CI 1.26-1.79) were associated with greater risk of developing any SMN. Asian ethnicity (compared to non-Hispanic White, IRR 0.61, 95% CI 0.43-0.87) and later calendar period of diagnosis (2003-2014 compared to 1990-2002, IRR 0.80, 95% CI 0.67-0.96) were associated with a lesser risk of developing any SMN.	Older age at diagnosis (30-39 vs. 15-19), female sex (vs. male), advanced stage at diagnosis (TMN stage II or III/IV vs. TMN stage I), radiation therapy (vs. no radiation therapy), Non-Hispanic White (vs. Asian/Pacific Islander)	Calendar period of diagnosis (2003-2014 vs. 1990-2002), Non-Hispanic Black, Hispanic (vs. Non-Hispanic White), Radiation (yes vs. no), Alkylating agents (yes vs. no), Anthracyclines (yes vs. no), Epipodphyllotoxin (yes vs. no), Platinums (yes vs. no)

Reference	Country of origin	Purpose	Data source	Cancer type	Diagnosis period	Age at diagnosis (years)	Survival entry date	Number of AYA participants	Follow-up time	Results	Statistically significant risk factors	Non-significant risk factors
Fidler, 2018 [26]	12 European countries*	To investigate the risk of subsequent primary bone cancers after childhood and adolescent cancer in 12 European countries.	PanCareSurFup	First primary malignancy: All cancers. SMN: bone cancers.	Varied by country	15-19	5-years post-diagnosis	11,472	Median 21.4 years	No risk factors reported.	None presented	NA
Zakaria, 2019 [20]	Canada	To quantify the risk and correlates of second cancer in Canadians diagnosed with a first cancer prior to age 20.	Death-linked Canadian Cancer Registry	Any primary malignancy except epithelial, basal and squamous skin cancer	1992-2014	15-19	61 days post-diagnosis	7,460	204,309 person-years	No risk factors reported.	None presented	NA
Reulen, 2020 [27]	12 European countries*	To estimate risk of specific digestive second primary neoplasms among 5-year cancer survivors of childhood cancer beyond age 40 years.	PanCareSurFup and linkage with population based national registries, other sources of national clinical data	Any primary malignancy except myelodysplastic syndrome, Langerhans cell histiocytosis, chronic myeloproliferative or lymphoproliferative disorder or a immunoproliferative disease	1940-2008 - Varies by country	15-19	5-years post-diagnosis	21,402	Median 16.3 years	No risk factors reported.	None presented	NA

Reference	Country of origin	Purpose	Data source	Cancer type	Diagnosis period	Age at diagnosis (years)	Survival entry date	Number of AYA participants	Follow-up time	Results	Statistically significant risk factors	Non-significant risk factors
Hodgkin lymphoma cohort												
Swerdlow, 2011 [28]	United Kingdom	To evaluate the risk of SMN in a large British cohort of HL patients treated with chemotherapy.	British National Lymphoma Investigation	HL	1963-2006 (varied by hospital)	25-34	At diagnosis	2,291	7,318 person-years (chemotherapy only group) and 12,123 person-years (chemotherapy plus radiotherapy group)	AYA cancer survivors (age 25-34) who underwent chemotherapy plus radiotherapy experienced a greater risk of second primary malignancies than survivors who underwent chemotherapy only (SIR 4.2, 95% CI 3.3-5.4 and AER 44.9 vs. SIR 2.9, 95% CI 1.9-4.4 and AER 21.6). This trend was true for the development of solid tumors, and NHL, but not for leukemia. For both treatments, the number of observed cases of SMN were significantly higher than what was expected in the general population.	Radiation in addition to chemotherapy (SIR higher than for chemotherapy alone)	No
Swerdlow, 2012 [29]	England and Wales	To examine breast cancer risks in relation to supradiaphragmatic radiotherapy in Britain.	Case notes, cancer registries, reports from clinicians, screening clinics, and patient	First Primary: HL. SPN: Breast Cancer	1956-2003	15-36	At diagnosis	4,767	84,725 person-years	SIR and AERs per 10,000 person-years were elevated in all five-year age groups, but were highest in the 15-19 age group (SIR 14.3, 95% CI 12.0-17.4; AER 58.6, 95% CI 47.7-71.1) and lowest in the 30-35 age group (SIR 2.4, 95% CI 1.8-3.0; AER 19.4, 95% CI 11.4-29.2).	Fewer years since diagnosis/treatment, younger age at first supradiaphragmatic RT (vs. older	No
Schaapveld, 2015 [30]	Netherlands	To investigate the long-term risk of a second cancer and changes in risk over time in a large cohort	Netherlands Cancer Registry	HL	1965-2000	15-34	5-years post-diagnosis	2,736	Median 19.1 years	For individuals who were aged 15-24 at the time of treatment for first HL, the SIR for experiencing any type of SMN was 8.4 (95% CI 7.5-9.5), with an associated AER of 111 per 10,000 person-years, compared to the general population. For individuals who were aged 25-34 at the time of treatment for first HL, the SIR for experiencing SMN was 5.0 (95% CI	Younger age at first treatment	No

Reference	Country of origin	Purpose	Data source	Cancer type	Diagnosis period	Age at diagnosis (years)	Survival entry date	Number of AYA participants	Follow-up time	Results	Statistically significant risk factors	Non-significant risk factors
		of HL survivors in the Netherlands.								4.4-5.6), with an associated AER of 118 per 10,000 person-years.		
Xavier, 2015 [31]	United States	To analyze SMN and survival outcomes of AYAs with early stage classical HL.	SEER	HL	1995-2010	15-39	6-months post-diagnosis	5,156	Median 97 months	At 150 months, the cumulative risk of SMN was 3.3% and 3.0% for people who had and had not received radiation therapy, respectively. The difference was not statistically significant (p=0.87). Among people treated with radiation therapy, carcinomas (59.5%), myelogenous leukemia/myelodysplastic syndrome (12.2%) and NHL (12.2%) were the most commonly developed SMNs. Among people not treated with RT, carcinomas (50%), NHL (17.3%) and melanomas (11.5%) were the most commonly developed SMNs.	None	Radiation therapy
Bhuller, 2016 [32]	Canada	To highlight specific long-term morbidity and mortality issues for TYA patients with HL.	Childhood, Adolescent and Young Adult Cancer Survivors (CAYACS) Research Program in British Columbia; cases identified from the British Columbia Cancer Registry	First Primary: HL. SPN: All cancers	1970-1999	15-24	5-years post-diagnosis	442	Median 19.6 years from diagnosis	All treatment modalities were associated with SMN. RT (HR=2.7, 95% CI 1.0-7.7) and female sex (HR=1.8, 95% CI 1.0-1.3) were associated with SMN in multivariate models. SIRs for SMN were statistically significant for individuals diagnosed between 1970-1979 and 1980-1989, but not 1990-1999.	Female sex (vs. male), Radiation therapy (vs. no RT), Earlier decades of diagnosis (SIRs significant)	No

Reference	Country of origin	Purpose	Data source	Cancer type	Diagnosis period	Age at diagnosis (years)	Survival entry date	Number of AYA participants	Follow-up time	Results	Statistically significant risk factors	Non-significant risk factors
van Eggermond, 2017 [33]	Netherlands	To assess long-term, site-specific risk of colorectal SMN associated with specific radiation fields and chemotherapy regimens in survivors of HL.	Dutch University Hospitals or Cancer Centres plus affiliated hospitals of the former population-based Eindhoven Cancer Registry	First primary malignancy: HL. SMN: colorectal cancer	1965-1995	25-34	5-years post-diagnosis	1,009	Median 22.9 years	No risk factors reported.	None presented	NA
Other tumor-specific cohorts												
Goldfarb, 2014 [34]	United States	To compare the tumor characteristics, treatment, and overall survival of SMN versus primary thyroid cancer in AYA patients.	National Cancer Database	Thyroid cancer	2004-2010	15-39	At diagnosis	41,062	Mean 35.5 months	Compared with cases of primary thyroid cancer, SMNs were more likely to occur in people who were White (OR=2.6, 95% CI 1.3-5.3), aged 35 to 39 years (OR=1.2, 95% CI 1.1-1.4), and whose tumors had tall/columnar cell histology (OR 2.19, 95% CI 1.5-3.2), were <1 cm (OR 1.50, 95% CI 1.3-1.7) or with multifocality (OR 1.17, 95% CI 1.05-1.3). Secondary thyroid cancers occurred less in females (OR=0.61, 95% CI 0.5-0.7), Hispanics (OR=0.78, 0.6-0.9), people aged 15 to 19 (OR=0.62, 95% CI 0.5-0.8) or 25 to 29 years (OR=0.71, 95% CI 0.6-0.8) at the time of SMN diagnosis.	White (vs. Hispanic), Older at diagnosis (35-39) (vs. 15-19 or 24-29), Male (vs. female)	None
Lee, 2014 [35]	United States	To describe the incidence, characteristics, and outcomes of	SEER	First Primary: osteosarcoma. SMN: all	1973-2010	21-39	6-months post-diagnosis	609	Median 4.2 years	No risk factors reported.	None presented	NA

Reference	Country of origin	Purpose	Data source	Cancer type	Diagnosis period	Age at diagnosis (years)	Survival entry date	Number of AYA participants	Follow-up time	Results	Statistically significant risk factors	Non-significant risk factors
		SMNs in long-term survivors of osteosarcoma.		cancers except osteosarcoma								
Sultan, 2019 [36]	United States	To estimate the incidence and type of second malignancies occurring in patients diagnosed with Ewing sarcoma family of tumors over a 33-year period.	SEER	First primary malignancy: Ewing sarcoma. SMN: All cancers excluding in situ tumors	1973-2005	20-39	At diagnosis	324	Mean 6.7 years	No risk factors reported.	None presented	NA
Abrahao, 2020 [37]	United States	To estimate the cumulative incidence of late effects at 10 years among HIV-uninfected and HIV-infected AYAs diagnosed with NHL during 1996-2012, who survived ≥2 years.	California Cancer Registry	NHL	1996-2012	15-39	2-years post-diagnosis	4,392 HIV-uninfected and 425 HIV-infected	Median 9.5 and 9.3 years for HIV-uninfected and HIV-infected, respectively	HIV-uninfected patients had a higher risk of second cancers if they were female (HR 1.52, 95% CI 1.09-2.11).	HIV-uninfected patients: Female (vs. male)	HIV-uninfected: Public/no health insurance (vs. private); Non-Hispanic White, non-Hispanic Black, non-Hispanic Asian/Pacific Islander (vs. non-Hispanic White); Low neighbourhood SES (vs. high); Lymphoblastic lymphoma, NK/T cell lymphoma, unspecifed (vs. B

Reference	Country of origin	Purpose	Data source	Cancer type	Diagnosis period	Age at diagnosis (years)	Survival entry date	Number of AYA participants	Follow-up time	Results	Statistically significant risk factors	Non-significant risk factors
												cell lymphoma); Hematopoietic cell transplant (yes vs. no); Year of diagnosis (2010-2014, 2005-2009, 2001-2004 vs. 1996-2000).
Gingrich, 2020 [38]	United States	To evaluate whether the development of medical conditions, 2 years after diagnosis, differed by socio-economic factors among AYA diagnosed with melanoma.	California Cancer Registry linked to data from the Office of Statewide Health Planning and Development (OSHPD)	Cutaneous melanoma	1996-2012	15-39	2-years post-diagnosis	8,259	NR: Results reported for 10-years post diagnosis	Compared to AYA 15-24 years of age, those 35-39 had higher risk of subsequent Melanoma (HR=1.3, 95%CI 1.1-1.7) and subsequent cancers other than melanoma (HR=1.3, 95% CI 1.0-1.6). Subsequent cancers were developed in males more than females (HR= 1.5, 95% CI 1.3-1.7), people with public/no insurance more than people with private insurance (HR= 2.3, 95% CI 1.9-2.8). Differences in ethnicity, neighbourhood SES and surgery (yes/no) were not statistically significant.	Older age at diagnosis(35-39 vs. 15-24), Male sex (vs. female), Public/No Health insurance (vs. private)	Hispanic or Other/Unknown (vs. Hon-Hispanic White), Surgery (vs. no surgery)
Muffly, 2020 [39]	United States	To evaluate late effects in AYAs diagnosed with ALL in California.	California Cancer Registry	ALL	1995-2012	15-39	3-years post-diagnosis	1,069	Median 8.2 years	Cumulative incidence of second cancer was higher among AYAs who had public or no health insurance (vs. private/military) (1.4, 95% CI 1.1-1.9) or who had received HCT (2.54, 95% CI 2.0-3.3).	Public or no health insurance (vs. private/military), Receipt of hematopoietic cell transplant (yes vs. no)	Hispanic, Asian/Pacific Islander, Black, Other/Unknown (vs. non-Hispanic White); Male sex (vs. female); Year of diagnosis 2001-2006 or 2007-2012 (vs. 1995-2000); Neighbourhood SES tertile (Low or Medium vs. High): Cranial

Reference	Country of origin	Purpose	Data source	Cancer type	Diagnosis period	Age at diagnosis (years)	Survival entry date	Number of AYA participants	Follow-up time	Results	Statistically significant risk factors	Non-significant risk factors
												irradiation (Yes vs. No)

Abbreviations: AER, absolute excess risk; ALL, acute lymphoblastic leukemia; AYA, adolescent and young adult; CI, confidence interval; CNS, central nervous system; HCT, hematopoietic cell transplant; HIV, human immunodeficiency virus; HL, Hodgkin lymphoma; HR, hazard ratio; NHL, non-Hodgkin lymphoma; NR, not reported; OR, odds ratio; SES, socioeconomic status; SIR, standardized incidence ratio; SMN, second malignant neoplasm; SPN, second primary neoplasm

Table S3. Risk factors associated with chronic conditions among adolescent and young adult cancer survivors by tumor group (n=34).

Reference	Country of origin	Purpose	Data source	Cancer type	Diagnosis period	Age at diagnosis (years)	Survival entry date	Number of AYA Participants	Follow-up	Results	Statistically significant risk factors	Non-significant risk factors
Mixed-cancer cohort												
Bradley, 2010 [50]	Canada	To describe the probability and frequency of hospital admission among childhood and adolescent cancer survivors over a 3-year period and compare to a population sample.	CAYACS linked to BC's health insurance plan Client Registry	Any primary malignancy	1970-1995 records linked from 1998-2000	15-19	5-years post-diagnosis	252	NR	No risk factors reported.	None presented	NA
Deyell, 2013 [51]	Canada	To determine if childhood and AYA cancer survivorship increased the likelihood of filling an antidepressant prescription.	CAYACS	Any primary malignancy	Diagnosed: 1970-1955 Linked to Insurance: 2001-2004	15-25	5-years post-diagnosis	1,237	NR	No risk factors reported.	None presented	Age at diagnosis
Zhang, 2014 [47]	Canada	To estimate the risk of late morbidity leading to hospitalization among young adult cancer survivors compared to the general population and to examine long term effects of demographic and disease-related factors on late morbidity.	British Columbia Cancer Registry linked to the Medical Service Plan	Any primary malignancy	1981-1999	20-24	5-years post-diagnosis	902	Mean 6.1 years	Survivors of tumors of the bone and CNS had the highest risk of hospitalization (RR= 2.2 (95% CI 0.88-5.49) and 2.09 (95% CI 0.96–4.56), respectively, but the increased risk was not statistically significant compared to the risk of survivors of leukemia. Females (vs. males) (RR=1.5, 95% CI 1.2-1.9), people who received chemotherapy, radiation and surgery (vs. chemotherapy only) (RR=1.8, 95% CI 1.1-3.1) and people living in Interior (RR=1.6, 95% CI 1.1-2.2) or Northern BC (RR=1.5, 95% CI 1.1-2.3) (vs. Vancouver	Female sex (vs. male sex), Living in Interior or Northern B.C. (vs. Vancouver coastal), Treatment modality (chemo, radiation and surgery combined vs. chemo only), Relapse status (Relapse vs. No relapse), Second cancer status (Second cancer	Living in Fraser or Island region of BC (vs. Vancouver coastal), Lowest quintile of SES (vs. Highest quintile of SES), Types of cancer (vs. leukemia), Calendar period of diagnosis (1986-1990, 1991-1995, 1996-1999 vs. 1981-1985), Treatment modality (radiation only,

Reference	Country of origin	Purpose	Data source	Cancer type	Diagnosis period	Age at diagnosis (years)	Survival entry date	Number of AYA Participants	Follow-up	Results	Statistically significant risk factors	Non-significant risk factors
										coastal) had higher risk of morbidity.	vs. No second cancer)	surgery only, chemo and radiation, chemo and surgery, radiation and surgery vs. chemo only)
Brewster, 2014 [52]	Scotland	To research acute and psychiatric hospital admission rates and length of stay in 5-year AYA survivors of cancer diagnosed before the age of 25.	National linked database that includes acute hospital dis1981-2003charge records, psychiatric hospital records, Scottish cancer registration and mortality records	Any primary malignancy	1981-2003	15-24	5-years post diagnosis	3,053	NR: Observed bed days for AYAs was 30,911	No risk factors reported.	None presented	NA
Kero, 2014 [49]	Finland	To investigate cardiac and vascular morbidity among early-onset cancer survivors.	Finnish Cancer Registry and Finnish Hospital Discharge Registry	Any primary malignancy	1975-1993	20-34	5-years post-diagnosis	9,401	NR	Risks varied by the treatment era and the type/site of cancer, with survivors of melanoma, renal, bone, soft tissue, colon, breast or thyroid cancer not having higher HRs for these cardiovascular outcomes. HR for several adverse outcomes were elevated among survivors of lymphoma and testicular malignancy.	Type of cancer, Earlier treatment era for cardiac arrhythmia, myocardial infarction/cardiac ischemia, atherosclerosis/brain vascular thrombosis (1975-1982 and 1983-1992 generally higher risk for cardiac outcomes)	Female vs. male

Reference	Country of origin	Purpose	Data source	Cancer type	Diagnosis period	Age at diagnosis (years)	Survival entry date	Number of AYA Participants	Follow-up	Results	Statistically significant risk factors	Non-significant risk factors
Kirchoff, 2014 [53]	United States	To report on hospitalizations among a population-based cohort of survivors of child and adolescent cancer from the Utah Cancer Registry in relation to a comparison cohort sampled to be similar on birth year and sex from the Utah Population Database.	UPDB; Utah Cancer Registry	Any primary malignancy	1973-2005	15-20	5-years post diagnosis	597	Mean 13.5 years	No risk factors reported.	None presented	NA
Rugbjerg, 2014 [48]	Denmark	To assess the rates of hospitalization for various cardiovascular conditions among 1-year survivors of AYA cancer and compare them with those of a cancer-free subset of the Danish population.	Danish Cancer Registry with linkage to the National Patient Register	Any primary malignancy	1943-2009	15-39	1-year post-diagnosis	43,153	Mean 15 years	HRR highest among survivors of leukemia (HRR=2.5, 95% CI 2.1-2.9), HL (HRR=1.7, 95% CI 1.6-1.9), brain (HRR=1.6, 95% CI 1.5-1.8). Survivors were also at risk of diabetes (HR=1.23, 95% CI 1.17-1.30), COPD (RR=1.23,95% CI 1.17-1.31) and chronic kidney disease (RR=2.3, 95% CI 2.04-2.60). Men were slightly more at risk for hospitalization for CVD than women, but confidence intervals overlapped (RR 1.34, 95% CI 1.30-1.39 vs. RR 1.28, 95% CI 1.25-1.32).	Type of cancer (leukemia, HL and brain tumor survivors at highest risk), Diagnosis in the earliest calendar years (1943-1959 vs. 1960-2009), Younger attained age (RR of cardiovascular hospitalization higher than more older attained ages), Fewer years since diagnosis (5 years)	Sex
van Laar, 2014 [54]	United Kingdom	To define the incidence and risk of cardiovascular late effects identified from inpatient hospital episode statistics (HES)	Yorkshire Specialist Register of Cancer in Children and Young People	Any primary malignancy except skin carcinom	1991-2006	15-29	5-years post-diagnosis	1,880	NR: at least 5 years	No risk factors reported.	None presented	NA

Reference	Country of origin	Purpose	Data source	Cancer type	Diagnosis period	Age at diagnosis (years)	Survival entry date	Number of AYA Participants	Follow-up	Results	Statistically significant risk factors	Non-significant risk factors
		among long-term survivors of cancer in young people.	linked to inpatient Hospital Episode Statistics	as and melanomas								
Ahomaki, 2015 [55]	Finland	To explore the risk of new psychiatric diagnoses in 5-year survivors of childhood and YA cancer compared with a sibling cohort.	Finnish Cancer Registry and Central Population Registry and Finish Hospital Discharge Registry	Any First primary Malignant Neoplasm. Excluded those with SMN	1975-2004	20-34	5-years post-diagnosis	9,543	NR	Compared to siblings, YA survivors had higher risk of organic memory/brain disorders (HR= 2.1; 95%CI 1.4–3.1); and mood disorders (HR=1.3; 95%CI 1.1–1.5). Females had significantly increased risk for neurotic/anxiety disorders (HR=1.6, 95% CI 1.2–2.1) compared to their siblings, whereas males did not. Radiotherapy did not explain the differences in psychiatric effects.	female sex (for anxiety/neurotic disorders)	Male sex (compared to siblings), Radiotherapy (vs. no radiotherapy), Decade of diagnosis (no difference in organic memory and brain disorders across 3 decades)
Asdahl, 2016 [56]	Denmark, Finland, Iceland, Norway & Sweden	To assess the frequency of gastrointestinal and liver late effects among childhood cancer survivors and compare this frequency with the general population.	Adult Life after Childhood Cancer in Scandinavia, national cancer registries in Denmark, Finland, Iceland, Norway and Sweden.	Any primary malignancy	1943-1958 to 2008-2010 (depending on the country)	15-19	1-year post-diagnosis	9,921	Median 10 years for cancer survivors and 15 years for comparison group	Survivors had 50% excess gastrointestinal or liver diseases compared to the general population (RR=1.5, 95% CI 1.4-1.6).	None presented	NA
Kero, 2016 [57]	Finland	To investigate the purchases of medications associated with metabolic syndrome (MetS) among 7,551 early onset	Finnish Cancer Registry linked to Drug	Any primary malignancy	1994-2004	20-34	At diagnosis	2,184	Median 9.5 years	Higher HR for purchasing anti-hypertensives (HR 1.5, 95% CI 1.3-1.8) , diabetes drugs (HR 1.6, 95% CI 1.1-2.2) and lipid-lowering drugs (HR=1.6, 95% CI 1.0-2.5) in YA cancer survivors	type of cancer	No

Reference	Country of origin	Purpose	Data source	Cancer type	Diagnosis period	Age at diagnosis (years)	Survival entry date	Number of AYA Participants	Follow-up	Results	Statistically significant risk factors	Non-significant risk factors
		cancer patients compared to siblings.	Purchase Registry							compared to siblings. Among specific cancer diagnosis groups, highest HR values for anti-hypertensives were found in YA ALL (HR 4.8, 95% CI 3.1-7.0) and myeloid leukemia (HR 3.4, 95% CI 2.2-5.1) patients. YA ALL patients showed strongest likelihood of purchasing diabetes drugs compared to siblings (HR 3.7, 95% CI 1.2-9.5)		
Chao, 2016 [58]	United States	To describe the epidemiology and risk factors for CVD (CVD) in survivors of AYA cancer.	Kaiser Permanente Southern California	Any primary malignancy	1998-2009	15-39	2-years post-diagnosis	5,673	Mean 4.4 years	For cancer survivors, incidence rate ratio for developing CVD was 2.37 (95% CI 1.9-2.9) compared to patients without cancer. Highest risk in leukemia (IRR=4.23, 95% CI 1.7-10.3) and breast cancer (IRR=3.63, 95% CI 2.4-5.5) survivors. Of the three cardiovascular risk factors examined, having diabetes (IRR=3.18, , 95% CI 1.9-5.5) or hypertension (IRR=3.67, 95% CI 2.4.-5.7) generally imposed a greater risk for CVD than dyslipidemia (IRR=1.79, 95% CI 1.1-2.9).	cardiovascular risk factors (diabetes, hypertension, dyslipidemia vs. not having these risk factors), type of cancer	No
Rugbjerg, 2016 [40]	Denmark	To examine relative an absolute excess risk for hospitalizations up to 34 years after diagnosis of AYA cancer compared to a comparison population.	Denmark using Danish Cancer Registry	Any primary malignancy except non-melanoma skin cancer	1943-2004	15-39	5-years post-diagnosis	33,555	Median 14 years	Highest risk of hospitalization by cancer diagnosis: Survivors of leukemia (RR 2.2, 95% CI 2.0-2.4), HL (RR 1.87, 95% CI 1.80-1.94) and brain cancer (RR 1.93, 95% CI 1.86-2.0).	Type of cancer (mainly leukemia, brain cancer, HL), Younger attained age (younger more at risk than older)	No

Reference	Country of origin	Purpose	Data source	Cancer type	Diagnosis period	Age at diagnosis (years)	Survival entry date	Number of AYA Participants	Follow-up	Results	Statistically significant risk factors	Non-significant risk factors
Bright, 2017 [45]	England & Wales	To investigate the risk of hospitalization for specific cerebrovascular events in survivors of all and specific types of cancer in AYAs.	Teenage and Young Adult Cancer Survivor Study, England and Wales Linked to Hospital Episode Statistics Data	Any primary malignancy	1971-2006	15-39	5-years post-diagnosis	178,962	Median 11.3 years	Survivors of CNS tumors (SHR=4.6, 95% CI 4.3-5.0), head and neck tumors (SHR=2.6, 95% CI 2.2-3.1) and leukemia (SHR=2.4, 95% CI 1.9-3.1) were at greatest risk of cerebrovascular events. Males were at higher risk than females (SHRmale=1.5, 95% CI 1.5-1.6 vs. SHRfemales=1.3, 95% 1.2-1.3). Younger survivors were more at risk than older survivors (SHR 15-19=3.6, 95% 3.0-4.2 vs. SHR 35-39=1.2, 95% CI 1.2-1.3).	Male sex (higher than for female sex), younger age at diagnosis (15-19 age group higher than 35-39 age group), younger attained age (15-19 age group higher than 35-39 age group), type of cancer, later decade of diagnosis (2000-2006 higher than preceding decades)	No
Jensen, 2018 [59]	Denmark	To investigate the lifetime risks of endocrine late effects of cancer and cancer treatment in adolescent and young adult cancer survivors compared to population-based cancer-free matched participants.	Danish Cancer Registry with linkage to the National Patient Register	Any primary malignancy	1977-2009	15-39	1-year post-diagnosis	32,584	Median 10 years for cancer survivors and 15 years for cancer-free	Type of cancer with highest RRs for any endocrine disease: Leukemia (RR 3.97, 95% CI 3.10-5.09), HL (RR 3.06, 95% CI 2.62-3.57), and brain cancer (RR 3.03, 95% CI 2.53-3.64). AYAs aged 15-19 had a higher risk of hospital contact for endocrine disease than AYAs aged 30-34 (RR=4.0, 95% CI 3.4-4.8 vs. RR=1.6, 95% CI 1.5-1.8). Females were more at risk of hospitalization for endocrine diseases compared to males (HR=1.9, 95% CI 1.6-2.1).	Younger attained age (16-19 and 20-29 lower than 40-49 and 50-59), type of cancer (compared to brain cancer, colon cancer, cervical cancer, malignant melanoma, ovarian cancer, breast cancer, NHL were less at risk, while leukemia was more at risk), female sex (vs. male sex), younger age at	No

Reference	Country of origin	Purpose	Data source	Cancer type	Diagnosis period	Age at diagnosis (years)	Survival entry date	Number of AYA Participants	Follow-up	Results	Statistically significant risk factors	Non-significant risk factors
											cancer diagnosis (reference group, 15-19, most at risk), later period of diagnosis (1990-2009 vs. 1975-1989), fewer years since diagnosis (1-4 years less than >20 years)	
Keegan, 2018 [46]	United States	To examine associations of SES characteristics with CVD after consideration for clinical factors among 2-year survivors of 14 common AYA cancers.	California Cancer Registry and State hospital discharge data	14 first primary AYA cancer	1996-2012	15-39	2-years post-diagnosis	79,176	Median 9 years	2.8% of survivors developed CVD. Highest 10-year CVD incidence in survivors of CNS cancer (7.3%, 95% CI 6.4-8.3), ALL (6.9%, 95% CI 5.2-8.7) AML (6.8%, 95% CI 5.3-8.7)) and NHL (4.1%, 95% CI 3.5-4.8). Risk of CVD was elevated for older AYAs (HR=2.6, 95% CI 2.1-3.2) (vs. younger), non-Hispanic Blacks (HR=1.6, 95% 1.3-1.8) (vs. non-Hispanic Whites), people with public/no health insurance (HR=1.8, 95% CI 1.6-2.0) (vs. military/private), people living in the lowest SES neighbourhoods (HR=1.6, 95% CI 1.4-1.9) (vs. highest). Risk of developing CVD was lower for females (HR=0.7, 95% CI 0.6-0.8) (vs. males). CVD incidence was higher for people who received chemotherapy (3.5% vs. 2.0% for radiation only) and a stem cell transplant (7.1% vs. 2.5% for no transplant). Diabetes, obesity, hypertension and dislipidemia	Non-Hispanic Black (vs. Non-Hispanic White), public or no health insurance (vs. private/military), residing in lower SES neighbourhoods (lowest quintile vs. highest quintile) (acute lymphoid leukemia, AML, bone sarcoma, central nervous system, breast, cervical, HL, melanoma, NHL, soft tissue sarcoma, testicular vs. thyroid), male sex (vs. female), chemotherapy or chemotherapy	Hispanic, Asian/Pacific Islander (vs. Non-Hispanic White)

Reference	Country of origin	Purpose	Data source	Cancer type	Diagnosis period	Age at diagnosis (years)	Survival entry date	Number of AYA Participants	Follow-up	Results	Statistically significant risk factors	Non-significant risk factors
										were also positively associated with the development of CVD.	and radiation as initial treatment, stem cell transplant (yes vs. no), type of cancer, older age at diagnosis (35-39 vs. 15-19), earlier year of diagnosis (1996-2000 vs. 2009-2012), no surgery (vs. yes), distant stage at diagnosis	
Krawczuk-Rybak, 2018 [60]	Poland	To analyze the general health status and epidemiology of organ late effects in the cohort of Polish childhood cancer survivors.	National database in Poland	Any primary malignancy	NR	15-18	2-years post-treatment	197	NR	No risk factors reported.	None presented	NA
Nathan, 2018 [61]	Canada	To evaluate risk factors for mental health problems in survivors of childhood cancer in Ontario, Canada.	Collection of Ontario health databases - Pediatric Oncology Group of Ontario's Networked Information System,	Any primary malignancy	1987-2008	15-18	5-years post diagnosis	537	Median 7.5 years	No risk factors reported.	None presented	NA
Ahomaki, 2019 [62]	Finland	To determine if cancer patients have an increased risk for first time anti-depressant purchases and, if so, if gender, type of cancer	Finnish Cancer Registry	Any primary malignancy	1994-2004	20-34	At diagnosis	4,598	NR	In both patients and siblings, females had a higher risk than males for AD purchases (p<0.001). Patients with leukemia and had the highest HR of AD purchase (HR =9.1, 95% CI 6.9-	Female sex, type of cancer (leukemia, CNS)	No

Reference	Country of origin	Purpose	Data source	Cancer type	Diagnosis period	Age at diagnosis (years)	Survival entry date	Number of AYA Participants	Follow-up	Results	Statistically significant risk factors	Non-significant risk factors
		or age at diagnosis affect the risk.								12.1) followed by CNS (HR=7.0 95% CI 5.6-8.7)		
Smith, 2019 [43]	United Kingdom	To quantify the incidence of hospitalizations due to respiratory disease in long-term survivors of childhood and young adult cancer, identify treatment related risk factors associated with risk of respiratory admissions, and describe patterns of readmission/ subsequent mortality.	Cancer registries in Yorkshire, England-Yorkshire Specialist Register of Cancer in Children and Young People linked to inpatient Hospital Episode Statistics	Any primary malignancy excluding skin carcinomas and melanomas	1990-2011	15-29	5-years post-diagnosis	2,627	NR	AYA cancer survivors diagnosed with carcinomas and leukemia were had the highest risk of respiratory admission (hospital rate ratio = 2.11 (95% CI 1.65-2.71) and 2.09 (95% CI 1.47-2.98), respectively)	Type of cancer (respiratory admissions for leukemia, carcinomas significantly higher than for CNS tumors, germ cell tumors)	NA
de Fine Licht, 2019 [44]	Denmark	To report of both hospital contacts and purchase of prescription drugs associated with MetS among survivors of AYA cancer and compare such events with those in a subset of the general population.	Denmark-Nationwide registries	Any primary malignancy	1994-2009	15-39	5-years post-diagnosis	11,822	Mean 8.3 years	Highest risks for hospitalization for diabetes in survivors of brain cancer (RR 2.9 95% CI 1.9-4.4) and HL (RR 2.4, 95% CI 1.4-4.0). These groups were also most likely to purchase a prescription for MetS-related diseases. Compared to the population cohort, men purchased more drugs for hyperlipidemia (standardized prescription rate ratio (SPRR)=1.2, 95% CI 1.1-1.3) and hypertension (SPRR=1.2, 95% CI 1.3-1.5) while women purchased more drugs against hyperlipidemia (SPRR=1.1, 95% CI 1.0-1.3) and hypertension (SPRR=1.2, 95% CI 1.1-1.3).	Type of cancer (varied by outcome), male sex (for both hospital contact and prescription drugs), Earlier age at diagnosis	Attained age

Reference	Country of origin	Purpose	Data source	Cancer type	Diagnosis period	Age at diagnosis (years)	Survival entry date	Number of AYA Participants	Follow-up	Results	Statistically significant risk factors	Non-significant risk factors
Anderson, 2020 [41]	United States	To examine the risk of inpatient hospitalizations among AYA cancer survivors compared with their siblings and the general population.	Utah Population Database	Any primary malignancy	1994-2015	15-39	2-years post-diagnosis	6,330	Median 4.9 years	Risk was most elevated for survivors of leukemia (HR=4.8, 95% CI:3.9-5.9), central nervous system (HR=3.5, 95% CI:2.8-4.2), colorectal cancers (HR=2.8, 95% CI 2.2-3.6), NHL (HR=2.8, 95% CI 2.2-3.5) and breast cancer (HR=2.4, 95% CI 2.0-2.8). The hospitalization patterns observed were similar when comparing AYA cancer survivors to their siblings. Males were hospitalized more than females, relative to their siblings (HR=1.8, 95% CI 1.6, 1.9).	Type of primary cancer (vs. matched population); male survivor (vs. matched population, HR for first hospitalization higher than that for females vs. matched population)	No
Bhandari, 2020 [63]	United States	To evaluate the association between BMI, treatment related toxicity and treatment outcome in children with solid tumors treated with cisplatin-containing regimens.	Los Angeles Children's Hospital	Solid tumors or non-hematologic malignancy	2009-2017-period of treatment	15-20	At diagnosis	54	Median 3.1 years post-diagnosis	No risk factors reported.	None presented	NA
Chao, 2020 [64]	United States	To evaluate the development of chronic comorbidities in survivors of AYA cancer who were members of Kaiser Permanente Southern California (KPSC) using a matched cohort design.	KPSC SEER-affiliated cancer registry	Any primary malignancy	2000-2012	15-39	2-years post diagnosis	6,778	Median 5.1 years	In adjusted analyses, use of trastuzumab was significantly associated with risk of cardiomyopathy or heart failure (IRR=8.1, 95% CI 2.5-25.5); head/brain/neck radiation was associated with the risk of stroke (IRR=3.5, 95% CI 5.9-37.2), thyroid disorders (IRR=3.1, 95% CI 2.2-4.4), diabetes (IRR=1.9, 95% CI 1.3-2.9). The risk of developing diabetes was significantly elevated for AYAs aged 30-39 (vs. 15-29) (IRR=2.4,	Risk factors varied by outcome. Cardiomyopathy/ heart failure: antibody - trastuzumab (>4,000 mg/m2 vs. none). Hearing loss: Head/brain radiation (Platinum > 450 mg/m2 or radiation >= 30	Sex (female vs. male) for cardiomyopathy/ heart failure, hearing loss, stroke, diabetes; age at diagnosis (older vs. younger) for cardiomyopathy/ heart failure, hearing loss, stroke, thyroid disorders;

Reference	Country of origin	Purpose	Data source	Cancer type	Diagnosis period	Age at diagnosis (years)	Survival entry date	Number of AYA Participants	Follow-up	Results	Statistically significant risk factors	Non-significant risk factors
										95% CI 1.7-3.5), but not for cardiomyopathy/heart failure, hearing loss, stroke or thyroid disorders. Females were more at risk than men for developing thyroid disorders (IRR=2.1, 95% CI 1.4-3.0). Non-Hispanic Blacks (IRR=2.3, 95% 1.4-3.5), Hispanics (IRR=2.2, 95% CI 1.6-3.0) and Asian/Pacific Islanders (IRR=1.8, 95% CI 1.1-2.8) all had a higher risk of diabetes than non-Hispanic Whites.	Gy vs. Platinum =< 450 g/m2 or radiation 0-29.9 Gy). Stroke: Head/brain/neck radiation (>= 30 Gy vs. 0-29.9 Gy). Thyroid disorders: Female sex (vs. male), Head/brain/neck radiation (>= 30 Gy vs. 0-29.9 Gy). Diabetes: Age Older age at cancer diagnosis (30-39 vs. 15-29), Asian/Pacific Islander, Hispanic, Non-Hispanic Black (vs. Non-Hispanic White), Head/brain/neck radiation (>= 30 Gy vs. 0-29.9 Gy)	race/ethnicity (vs. Non-Hispanic White) for cardiomyopathy/heart failure, hearing loss, stroke, thyroid disorders
Yu, 2020 [65]	United States	To determine the association between pubertal development and post pubertal gonadal function in childhood cancer survivors.	Walter Reed National Military Medical Center childhood cancer survivor clinic registry and medical records	Any malignancy	1985-2010-period of follow-up	15-18	At completion of cancer treatment	7	Mean 9 years after cancer treatment	No risk factors reported.	None presented	NA

Reference	Country of origin	Purpose	Data source	Cancer type	Diagnosis period	Age at diagnosis (years)	Survival entry date	Number of AYA Participants	Follow-up	Results	Statistically significant risk factors	Non-significant risk factors
Suh, 2020 [42]	United States & Canada	To describe chronic health conditions and all-cause and specific mortality among survivors of early-adolescent and YA cancer.	Childhood Cancer Survivorship Study	Leukemia, CNS malignancy, HL, NHL, Wilms tumor, Neuroblastoma, Soft-tissue sarcoma, and Bone cancer	1970-1999	15-20	5-years post-diagnosis	4,082	Median 16.9 years	Cardiac conditions were higher among people who received chest radiation therapy of >/= 35 Gy (vs. none) (HR=3.3, 95% CI 2.4-4.6) and lower among females (vs. males) (HR=0.6, 95% CI 0.5-0.7). Endocrine conditions were higher among people who had total body radiation therapy (HR=3.3, 95% CI 1.4-7.8) or chest/neck radiation therapy (HR=3.9, 95% CI 2.6-5.7) (vs. none), and females (vs. males) (HR=3.2, 95% CI 2.4-4.3). Pulmonary conditions were higher among people who received chest radiation therapy >/= 35 Gy (vs. none) (HR=3.1, 95% CI 1.6-6.2).	Female sex (vs. male) for endocrine conditions; male sex (vs. female) for cardiac conditions; radiation (yes vs. no)	Sex (female vs. male) for pulmonary conditions, Race/ethnicity (Hispanic or "non-White" vs. non-Hispanic White)
Hodgkin lymphoma cohort												
van Nimwegen, 2015 [66]	Netherlands	To examine excess risk of CVD in HL up to 40 years after treatment relative to the risk in the general population.	Dutch university hospitals or cancer centers	HL	1965-1995	18-39	5-years post diagnosis	1,864	Median 20.3 years	Younger age at treatment was significantly associated with greater risk of both CHD (<18 at treatment: SIR=8.8, 95% CI 6.3-12.3; 25-29 at first treatment: SIR=4.1, 95% CI 3.3-5.1) and HF (<18 at treatment: SIR=38.9, 95% CI 25.2-57.4; 25-29 at first treatment: SIR=10.4, 95% CI 7.5-14.2).	Younger age at treatment	No
Keegan, 2018 [67]	United States	To examine the impact of race/ethnicity, neighbourhood SES and health insurance on the occurrence of medical	California Cancer Registry linked to hospitalization data	HL	1996-2012	15-39	2-years post-diagnosis	5,085	Median 9.5 years	In multivariable analyses, Black HL survivors were more likely to experience circulatory system (HR=1.6, 95% CI 1.2-2.1) and endocrine (HR=1.4, 95% CI 1.1-1.8), but not respiratory, liver, kidney or thyroid diseases	Race/ethnicity (Black vs. Non-Hispanic White), residing in low SES neighbourhoods (vs. high),	Race/ethnicity (Hispanic or Non-Hispanic Asian/Pacific Islander vs. White), earlier year of diagnosis

Reference	Country of origin	Purpose	Data source	Cancer type	Diagnosis period	Age at diagnosis (years)	Survival entry date	Number of AYA Participants	Follow-up	Results	Statistically significant risk factors	Non-significant risk factors
		conditions in AYA HL survivors.								compared to non-Hispanic Whites. Having public or no health insurance (vs. private/military) was associated with a higher risk of conditions ranging from circulatory (HR=1.4, 95% 1.2-1.7) to chronic kidney disease (HR=1.8, 95% CI 1.3-2.5), but not for thyroid conditions. Low neighbourhood SES (vs. high) was associated with an increased risk of respiratory diseases (HR=1.3, 95% CI 1.1-1.5). Circulatory system, respiratory system and hypothyroidism occurred more frequently among AYAs who received RT alone.	public/no insurance (vs. private/military), stem cell transplant (yes vs. no), disease stage (III/IV generally more at risk for medical conditions than I/II)	(2001-2004, 2005-2008, 2009-2012 vs. 1996-2000)
Other tumor-specific cohort												
Bhuller, 2016 [32]	Canada	To highlight specific, long-term morbidity and mortality issues for TYA patients with HL.	Childhood, Adolescent and Young Adult Cancer Survivors (CAYACS) Research Program in British Columbia; cases identified from the British Columbia Cancer Registry	First primary malignancy: HL. SMN: Any cancer based on ICDO-3 with behaviour code 3 or higher	1981-1999	15-24	5 years post-diagnosis	281	Median 19.6 years post diagnosis	Risk of morbidity requiring hospitalization is 2.5 fold for survivors diagnosed between 1996–1999 compared to survivors diagnosed between 1981–1985.	more recent eras of therapy (1996-1999 vs. 1981-1985)	Sex (Female vs. Male), Age at diagnosis 20-24 vs. 15-29), Rural vs. urban, region of residence (Interior, Fraser, Island, Northern vs. Vancouver coastal), Quintile of SES (Lowest vs. highest), Chemotherapy (Yes vs. No), Radiation (Yes vs. No), Surgical related therapy (Yes vs. no),

Reference	Country of origin	Purpose	Data source	Cancer type	Diagnosis period	Age at diagnosis (years)	Survival entry date	Number of AYA Participants	Follow-up	Results	Statistically significant risk factors	Non-significant risk factors
												Having late relapse (Yes vs. No), Having SMN (Yes vs. No)
Gunn, 2015 [68]	Finland	To evaluate late morbidity of survivors diagnosed in AYA survivors of brain tumors.	Finnish Cancer Registry and Hospital Discharge Registry	Brain tumors	1970-2004	16-24	5-years post-diagnosis	315	Median 11.5 years	Diseases of the nervous system (HR=3.3, 95% CI 1.8-6.2) were elevated in survivors who had received irradiation treatment compared to survivors who did not.	Radiation therapy (vs. no radiation)	No
Abrahao, 2020 [37]	United States	To estimate the cumulative incidence of late effects at 10 years among HIV-uninfected and HIV-infected AYAs diagnosed with NHL during 1996-2012, who survived ≥2 years.	California Cancer Registry	NHL	1996-2012	15-39	2-years post-diagnosis	4,392 HIV-uninfected and 425 HIV-infected	Median 9.5 and 9.3 years for HIV-uninfected and HIV-infected, respectively	For HIV-uninfected survivors, having public/no insurance (vs. private) was associated with an increased risk of all studied conditions, except avascular necrosis. HRs ranged from 1.5 (95% CI 1.3-1.8) for endocrine conditions, to 2.4 (95% CI 1.5-3.8) for neurologic conditions. Race/ethnicity was significantly associated with an increased risk of renal disease for Black (HR=1.9, 95% CI 1.0-3.6) and Hispanic (HR=1.7, 95% CI 1.1-2.7) survivors (vs. White). Low neighbourhood SES (vs. high) was associated with higher risk of cardiovascular 9HR=1.4, 95% CI 1.2-1.7), respiratory (HR=1.4, 95% CI 1.0-1.8) and endocrine diseases (HR=1.4, 95% CI 1.2-1.6).	HIV-uninfected survivors: public or no health insurance (vs. private), residence in lower SES neighbourhood (vs. high), receipt of a hematopoietic stem cell transplant (Yes vs. no)	HIV-uninfected survivors: Sex (Female vs. Male for cardiovascular, respiratory, liver/pancreatic, endocrine, neurologic, avascular necrosis), Non-Hispanic Black, Hispanic, Non-Hispanic Asian/Pacific Islander (vs. Non-Hispanic White), Year of diagnosis (2001-2004, 2005-2009, 2010-2014 vs. 1996-2000)
Gingrich, 2020 [38]	United States	To evaluate whether the development of medical conditions, 2 years after diagnosis, differed by socio-economic factors	California Cancer Registry linked to data from the Office of	Cutaneous melanoma	1996-2012	15-39	2-years post-diagnosis	8,259	NR	Compared to AYA 15-24, a higher risk of endocrine disorders was found among AYA 35-39 (HR:1.4, 95%CI 1.1-1.7); and higher risk of lymphedema was found among	Risk factors varied by condition. Older age at diagnosis (35-39 vs. 15-24) for endocrine	Race/ethnicity (Hispanic, "Other" vs. Non-Hispanic White),

Reference	Country of origin	Purpose	Data source	Cancer type	Diagnosis period	Age at diagnosis (years)	Survival entry date	Number of AYA Participants	Follow-up	Results	Statistically significant risk factors	Non-significant risk factors
		among AYA diagnosed with melanoma.	Statewide Health Planning and Development							AYA 30-34 (HR:1.3, 95%CI 1.02-1.3) and among AYA 35-39 (HR:1.7, 95%CI 1.4-2.1)	disorders, lymphedema; Male sex (vs. female); Public/no insurance (vs. private); Low neighbourhood SES (vs. high) for hematologic disorders, cardiac disease, diabetes, endocrine disorders, lymphedema, VTW, autoimmune disorders, infection/sepsis;	surgery (no vs. yes)
Muffly, 2020 [39]	United States	To evaluate late effects in AYAs diagnosed with ALL (ALL) in California.	California Cancer Registry	ALL	1995-2012	15-39	3-years post-diagnosis	1,069	Median 8.2 years IQR: 4.9-13.8	Having public or no health insurance (compared to private/military) and receipt of hematopoietic cell transplantation were associated with increased HR of all late effects. AYAs diagnosed between 2007-2012 were more likely to developed endocrine diseases (HR 1.48, 95% CI 1.11-1.98), while AYAs diagnosed between 2001 and 2006 were less likely to have an avascular event (HR 0.70, 95% CI 0.52-0.95) relative to AYAs diagnosed from 1995-2000.	Public or no health insurance (vs. private/military), receipt of hematopoietic cell transplant (yes vs. no)	Race/ethnicity (Hispanic, Asian/Pacific Islander, Non-Hispanic, Black vs. Non-Hispanic White), Sex (male vs. female), Era of diagnosis (2007-2012, 2001-2006 vs. 1995-2000), Neighbourhood SES tertile (low vs. high), cranial irradiation (yes vs. no)
Perisa, 2020 [69]	United States	To examine clinical features at presentation, treatment factors,	Nationwide Children's Hospital	Ewing Sarcoma	1990-2017	15-39	At diagnosis	45	Median 98 months	No risk factors reported.	None presented	NA

Reference	Country of origin	Purpose	Data source	Cancer type	Diagnosis period	Age at diagnosis (years)	Survival entry date	Number of AYA Participants	Follow-up	Results	Statistically significant risk factors	Non-significant risk factors
		toxicities, and outcomes in Ewing sarcoma AYA survivors compared with pediatric patients.	Columbus, Ohio									

Abbreviations: AER, absolute excess risk; ALL, acute lymphoblastic leukemia; AYA, adolescent and young adult; CI, confidence interval; CNS, central nervous system; CVD, cardiovascular disease; HIV, human immunodeficiency virus; HL, Hodgkin lymphoma; HR, hazard ratio; IRR, incidence rate ratio; MetS, metabolic syndrome; NHL, non-Hodgkin lymphoma; NR, not reported; OR, odds ratio; RR, rate ratio; SBDR, standardized bed day ratio; SES, socioeconomic status; SHR, standardized hospitalization ratio; SIR, standardized incidence ratio; SMN, second malignant neoplasm; SPN, second primary neoplasm; YA, young adult

Table S4. Risk factors associated with late mortality among adolescent and young adult cancer survivors by tumor group (n=54).

Reference	Country of origin	Purpose	Data source	Cancer type	Diagnosis period	Age at diagnosis (years)	Survival entry date	Number of AYA participants	Follow-up time	Results	Statistically significant risk factors	Non-significant risk factors
Mixed-cancer cohort												
Garwicz S, 2012 [71]	Denmark, Finland, Iceland, Norway & Sweden	To evaluate late and very late mortality, and patterns of causes of death, in 5-year survivors after childhood and adolescent cancer in cases diagnosed during four decades in the five Nordic countries.	Nordic Childhood Cancer Cohort	Any primary malignancy	1960-1999	15-19	5-years post-diagnosis	NR: Number of AYAs (15-19) in the age stratified analysis not reported	NR: 331,384 person-years. Median follow-up 13.0 years for the entire cohort starting at 5 years		None presented	NA
Prasad P, 2012 [72]	Finland	To evaluate non-malignant mortality patterns (specifically circulatory and respiratory disease deaths) among five-year survivors of AYA cancers in a large, Finnish population-based cohort, and to compare these rates to those in the general population.	Finnish Cancer Registry linked to National population Registry	Any solid tumor or hematological malignancy	1966-1999	15-34	5-years post-diagnosis	6,297	Median 15.9 years	SMR for all causes of death: for ages 15-19 (9.2, 95% CI 7.8-10.6) and for ages 20-34 (5.8, 95% CI 5.4-6.2). SMR for death due to circulatory disease: for diagnosis of HL, 8.4 (95% CI 3.1-18.2) for ages 15-19 and 6.5 (95% CI 4.6-8.9) for ages 20-34. For diagnosis of NHL, 21.8 (95% CI 7.1-50.8) for ages 15-19 and 3.3 (95% CI 1.4-6.5) for ages 20-34. For CNS tumor, 1.2 (95% CI 0.03-6.6; non-significant) for ages 15-19 and 3.2 (95% CI 1.3-6.5) for ages 20-34.	Type of cancer (only for mortality due to circulatory disease which reports stratified by age. Only 3 types of cancers evaluated HL, NHL, CNS. CNS not significant SMR for 15-29 but significant for 20-34); Younger age at diagnosis (15-19 vs. 20-34)	None stratified by Age

Reference	Country of origin	Purpose	Data source	Cancer type	Diagnosis period	Age at diagnosis (years)	Survival entry date	Number of AYA participants	Follow-up time	Results	Statistically significant risk factors	Non-significant risk factors
Zhang, 2012 [18]	Canada	To assess long-term risks of overall and cause-specific mortality and SMN among survivors of young adult cancer compared to the risk in the general British Columbian population and evaluate the effects of demographic and clinical factors on risk.	Childhood, Adolescent and Young Adult Cancer Survivors Research Program in British Columbia; cases identified fro the British Columbia Cancer Registry	Any primary malignancy	1970-1995	20-24	5-years post-diagnosis	1,248	21,711 person-years	Highest SMR for CNS tumor survivors (SMR 23.6, 95% CI 15.1-35.1; AER 27.8); lowest SMR for germ cell tumors (SMR 2.1 95% CI, 1.0-4.0; AER 1.6). Higher SMR between 5-19 years follow-up time (SMR 27.8, 95% CI 22.5-33.8; AER 19.4) than 20+ years follow-uptime (SMR 2.0, 95% CI 1.4-2.7; AER 1.2). Females at higher risk than males (SMR=6.3; 95% CI, 4.8–8.3; AER 3.8 vs SMR=5.6; 95% CI, 4.5–6.9; AER 7.1). Radiation therapy was associated with a 2-fold increase in risk of all-cause mortality (HR=2.0, 95% CI 1.3-3.1).	Type of primary cancer (All significant SMR but CNS highest AER than the others. Lowest AER for Germ cell tumors), chemotherapy (No higher AER for males; but lower for females), radiation treatment (yes higher AER than no), surgery (no higher AER than yes), shorter follow-up time (5-19 higher AER than 20+), most recent diagnosis period (1990-1995 higher AER), sex (female worst for overall mortality)	No
Haggar F, 2013 [70]	Australia	To estimate relative survival ratios and model excess mortality from malignancies in	Western Australian Data	Any primary malignancy	1982-2004	15-39	At diagnosis	10,266	Median 8.2 years	Overall 5-year relative survival rates for AYAs diagnosed with any cancer in the most recent diagnostic period (2000-2004) was 0.84 (95% CI 0.82–0.86) in males and 0.86 (95% CI	type of cancer, sex (typically male sex worse), era of diagnosis	Some not significant reported for cancer specific: Calendar

Reference	Country of origin	Purpose	Data source	Cancer type	Diagnosis period	Age at diagnosis (years)	Survival entry date	Number of AYA participants	Follow-up time	Results	Statistically significant risk factors	Non-significant risk factors
		AYAs, highlighting differences in outcomes according to gender, age, socio-economic status, geographic location and calendar period of diagnosis.	Linkage System							0.85–0.88) in females. Diagnoses of melanoma, germ cell tumors had good prognoses over 5 years (relative survival ratios=0.96 to 1.00). Diagnoses of leukemia and CNS malignancies had poorer prognoses over 5 years (SRS=0.5 to 0.6). For all AYA cancers combined, excess mortality increased with increasing age at diagnosis. Survival for all cancers combined higher for females vs. males (HR =0.66, 95% CI 0.62–0.71). Aboriginal AYAs experienced a higher excess mortality (HR=1.47, 95% CI 1.23–1.76) compared with non-Aboriginal AYAs. Increased mortality for AYAs living in rural and remote areas compared with those living in urban areas (HR=1.13, 95% CI 1.04–1.23), but the only subgroup in which this was true was for carcinomas. Increased mortality with declining SES for all cancers combined (HR, lowest vs. highest quartile: 1.14, 95% CI 1.04-1.26). Mortality was higher for older AYA cancer survivors (30-39 vs. 15-19: HR=1.4, 95% CI 1.2-1.6).	(except for CNS and soft tissue sarcoma; Ref 1985-1989), older age at diagnosis (30-39 vs 15-19), Aboriginal status (vs non-Aboriginal), living in rural/remote area (vs Urban areas), lower SES(lowest vs highest quartile-ref)	period of diagnosis for CNS and soft-tissue sarcoma (ref 1985-1989); Sex for CNS (ref male); Aboriginal status for Leukemia, Lymphoma, CNS, Soft-tissue, and Melanoma (ref non-aboriginal); Age at diagnosis (30-39 vs 15-19 not significant for Soft tissue, germ cell, and melanoma
Kero A, 2014 [49]	Finland	To investigate the cause-specific late mortality in 5-year survivors of childhood and young adulthood cancer diagnosed between 1966 and 2004 in Finland	Finnish Cancer Registry	Any primary malignancy except carcinoma in situ lesion of the skin	1996-2004	20-34	5-years post-diagnosis	11,417	NR: 6,000 individuals had follow-up beyond 30 years	Highest SMR (all-cause mortality) for survivors of ALL (SMR =14.2, 95% CI 7.4–20.9) and CNS tumor (SMR=12.3, 95% CI 11.2–13.4).	Type of cancer for overall cause of mortality (higher SMR for CNS compared to HL and NHL)	Type of cancer: Not significant SMR for CNS for cardiac ischemia cause of death; and NHL for

Reference	Country of origin	Purpose	Data source	Cancer type	Diagnosis period	Age at diagnosis (years)	Survival entry date	Number of AYA participants	Follow-up time	Results	Statistically significant risk factors	Non-significant risk factors
		using national population health registries.										respiratory cause of death.
Chao C, 2016 [58]	United States	To describe the epidemiology and risk factors for CVD in survivors of adolescent and young adult (AYA) cancer.	Kaiser Permanente Southern California	Any primary malignancy	1998-2009	15-39	2-years post-diagnosis	5,673	Mean 4.4 years	Higher all-cause mortality in cancer survivors who developed CVD compared to survivors without CVD (HR 10.9, 95% CI 8.1-14.8). Compared to those without CVD, survivors who developed CVD had lower 5 (0.67 with CVD vs 0.92 without CVD) and 10 years (0.55 vs. 0.90) survival after diagnosis.	CVD (vs. without CVD)	No
Henrique L, 2016 [73]	Brazil	To identify factors associated with early mortality from cancer in adolescents and young adults in a reference institution for oncology treatment in Santa Catarina, Brazil.	The information system of hospital cancer records (Sistema de Informações de Registro Hospitalar de Câncer),	Neoplasia excluding primary tumors in the CNS	2002-2013	15-29	At diagnosis	889	NR: "Cohort of 12 years"	Adjusting for neoplasia and sex: Higher risk of dying in individuals with non-hematological neoplasia (solid tumors) compared with individuals diagnosed with leukemias and lymphomas (HR: 1.47, 95%CI: 1.12–1.93). Adjusting for neoplasia and age: Compared with individuals diagnosed with leukemias and lymphomas, individuals diagnosed with non-hematological neoplasia had greater risk of death (HR: 1.51, 95%CI: 1.15–1.99).	male sex (vs. Female), younger age at diagnosis (15-24 vs 25-29), fewer years in school, longer time between diagnosis and treatment, non-hematological neoplasia (vs. hematological)	No
Henson K, 2016 [74]	England & Wales	To investigate long-term risk of cardiac mortality among 5-year survivors of TYA cancer.	Teenage and Young Adult Cancer Survivor Study	Any primary malignancy	1971-2006	15-39	5-years post-diagnosis	200,945	Mean of 14.3 years	2016 survivors died of cardiac disease. The SMR for all cardiac diseases was 1.4 (95% CI 1.3-1.4). SMR decreases with age at diagnosis; highest SMR for all cardiac diseases for individuals diagnosed at 15-19 (SMR=4.2, 95% CI 3.4-5.2) and lowest for individuals diagnosed at 35-39 (SMR=1.2, 95% CI 1.1-1.3). Compared to the general	For all cardiac deaths: Type of first cancer HL-referent(vs. Breast, testicular, cervix, melanoma, CNS, NHL, thyroid, gastrointestinal	Type of cancer: Not significant HL-ref (vs. AML). Female sex (vs. male for CNS); Younger age at diagnosis (15-19 vs. 20-24, 25-29, 30-24,35-39, for NHL,

Reference	Country of origin	Purpose	Data source	Cancer type	Diagnosis period	Age at diagnosis (years)	Survival entry date	Number of AYA participants	Follow-up time	Results	Statistically significant risk factors	Non-significant risk factors
										population, higher SMR were observed for survivors of HL (SMR: 3.8; 95% CI, 3.5–4.2), AML (SMR: 2.7; 95% CI, 1.6–4.4), genitourinary cancers other than bladder cancer (SMR: 2.0; 95% CI, 1.6–2.5), NHL (SMR: 1.7, 95% CI, 1.5–2.1), lung cancer (SMR: 1.7; 95% CI, 1.2–2.4), leukemia other than acute myeloid (SMR: 1.6, 95% CI, 1.0–2.4), central nervous system tumor (SMR: 1.4; 95% CI, 1.1–1.6), cervical cancer (SMR:1.3; 95% CI, 1.1–1.5), and breast cancer (SMR: 1.2; 95% CI, 1.1–1.4).	, soft tissue sarcoma, ovary, bladder, kidney and GU tract, head and neck, Leukemia (except ALL), bone tumor, lung) , younger age at diagnosis 15-19(vs 20-24, 25-29, 30-34, 35-39). female sex (vs. Male for HL and NHL).	CNS), decade of cancer diagnosis (1980-89, 1990-99, 2000+ vs. 15-19). Attained age (40-49, 50-59, 60+ vs. 20-39)
Berkman A, 2017 [75]	United States	To assess racial differences in risks of any death and CVD death by common cancer type among Black and White survivors of childhood and AYA (diagnosed before age 34) cancers at 5, 10, and 20 years post-diagnosis.	SEER	Any primary malignancy	1973-2011	15-34	At diagnosis	135,705	Median 15.1 years (until all-cause death)	Among AYA cancer survivors, Blacks were more likely than Whites to die from any cause at 10 (HR 1.91, 95% CI 1.85, 1.97) and 20 years (HR 1.89, 95% CI 1.84, 1.95). Survivors of germ cell cancer (HR 2.03, 95% CI 1.66, 2.48), melanoma (HR 1.89, 95% CI 1.14, 3.14), and HL (HR 1.63, 95% CI 1.44, 1.84) had the highest risk at 20 years. Risk of death was significantly higher for Blacks than Whites for all cancers but thyroid. For CVD deaths, specifically, Black survivors of AYA leukemias (HR 1.68, 95% CI: 1.06, 2.65), NHL (HR 3.25, 95% CI 1.56, 6.77), thyroid (HR 14.31, 95% CI 3.44, 59.45), melanoma (HR 2.42, 95% CI 1.89, 3.10) and other cancers	Any-cause of death: Black race/ethnicity (vs White), Type of cancer Black race (vs. White, Leukemias, HL, NHL, CNS, Germ Cell, Melanoma, Other for both 10-and 20-year survival).	No

Reference	Country of origin	Purpose	Data source	Cancer type	Diagnosis period	Age at diagnosis (years)	Survival entry date	Number of AYA participants	Follow-up time	Results	Statistically significant risk factors	Non-significant risk factors
										(HR 2.54, 95% CI 2.13, 3.05) had a higher risk at 20 years.		
Anderson C, 2018 [76]	United States	To estimate 5-year relative survival for AYAs with cancer, at diagnosis and for each additional year survived (i.e. conditional five-year relative survival) up to 25 years post-diagnosis.	SEER-9	Any primary malignancy except Kaposi sarcoma	1973-2009	15-39	At diagnosis	205,954	NR: Analysis was for 7 years	At 7 years, relative survival of AYA cancer survivors exceeded 95% compared with the general population. Greater relative survival for patients diagnosed in 1988-2009 compared to those diagnosed 1973-1987. Survival improvements over time were noted for most cancers. There was no difference in conditional 5-, 10- or 20-year survival between males and females when all cancers were reported together, but females had somewhat better survival outcomes for most cancer sites, particularly in the years closest to diagnosis.	male sex (vs female most sites), older age at diagnosis (30-39 vs 15-29), type of cancer (CNS, female breast, HL, and Leukemia had excess mortality risk), earlier era of diagnosis (1973-1987 vs 1988-2009)	No. States that very little excess mortality compared to the general population observed in those surviving at least 7 years after diagnosis
Fidler M, 2018 [77]	England & Wales	To investigate the risk of long-term respiratory mortality among 5-year cancer survivors diagnosed before age 40 years using the British Childhood Cancer Survivor Study and Teenage and Young Adult Cancer Survivor Study.	British Childhood Cancer Survivor Study and Teenage and Young Adult Cancer Survivor Study	Any primary malignancy	1971-2006 for TYACCS	15-39	5-years post-diagnosis	200,945	Mean 19.3 years	The proportion of deaths attributable to respiratory causes was highest among lung cancer survivors at 10%, followed by head and neck cancer survivors and cervical cancer survivors at 6% each. Pneumonia deaths were higher among males than females and survivors of lung cancer, CNS tumors and head and neck cancers.	Type of cancer (Lung, Leukemia (except AML), head and neck, and CNS greatest AER of overall respiratory deaths; Lung, CNS, Head and Neck greatest AER for pneumonia, Lung for chronic lower respiratory disease), male sex (vs Female)	Not significant AER: Type of cancer (Breast, Testicular, Thyroid, Gastrointestinal, soft tissue sarcoma, Bladder, Other genitourinary, for all respiratory diseases studied), AML (all except other respiratory illness), Ovary, Leukemia

Reference	Country of origin	Purpose	Data source	Cancer type	Diagnosis period	Age at diagnosis (years)	Survival entry date	Number of AYA participants	Follow-up time	Results	Statistically significant risk factors	Non-significant risk factors
											greater AER for overall respiratory deaths, and pneumonia, Female sex (vs Male) greater AER for fibrosis, higher attained age 60+ (vs 20-29) for overall, Chronic lower respiratory disease, for pneumonia and pneumonitis, early treatment era <1970 (vs 1990-2006) for pneumonia and chronic lower respiratory death; older age at diagnosis (Higher AER as age at diagnosis increased) for pneumonia, and Chronic lower respiratory disease	(except AML), other, bone tumor, melanoma (all except pneumonia), Cervical (all except pneumonia and chronic lower respiratory disease), CNS (Chronic respiratory disease and fibrosis) NHL (Chronic lower respiratory disease, Fibrosis and pneumonitis), head and neck (fibrosis and other respiratory disease), HL (pneumonitis)); Age at diagnosis (15-19 (for pneumonitis), 25-29 (all except pneumonia and other respiratory illness), 30-34 (chronic lower

Reference	Country of origin	Purpose	Data source	Cancer type	Diagnosis period	Age at diagnosis (years)	Survival entry date	Number of AYA participants	Follow-up time	Results	Statistically significant risk factors	Non-significant risk factors
												respiratory disease and pneumonitis), 35-39 (Fibrosis and pneumonitis)); Treatment Era 1970-1979 (Fibrosis and other respiratory illness), 1980-1989 (Pneumonitis), 1990-1999 (chronic lower respiratory disease and pneumonitis), 2000-2006 (all except pneumonia and other respiratory illness); Attained age 20-29 (all except pneumonia and other respiratory illness), 50-59 (pneumonitis), 60+ (Fibrosis and other respiratory illness)

Reference	Country of origin	Purpose	Data source	Cancer type	Diagnosis period	Age at diagnosis (years)	Survival entry date	Number of AYA participants	Follow-up time	Results	Statistically significant risk factors	Non-significant risk factors
Hayek S, 2018 [23]	Israel	To estimate the risk of second primary neoplasm and long-term mortality among 5+year childhood cancer survivors relative to their counterparts in the general population.	Israel National Cancer Registry	Any primary malignancy	1980-2007	15-19	5-years post-diagnosis	1,765	Age-specific follow-up NR		None presented	NA
Keegan T, 2018 [46]	United States	To examine associations of SES characteristics with CVD after consideration for clinical factors among 2-year survivors of 14 common AYA cancers.	California Cancer Registry and State hospital discharge data	14 First primary AYA cancers: female breast carcinoma, melanoma, thyroid carcinoma, testicular cancer, HL, NHL, soft tissue sarcoma, bone sarcoma, colorectal cancer, CNS cancer, cervical cancer and ovarian cancer	1996-2012	15-39	2-years post-diagnosis	79,176	Median 9 years	There was an 8-fold or higher increased risk of death among AYAs who developed CVD compared to those who did not. HR was as high as 37 (95% CI 28.2-48.6) for melanoma and as low as 8 (95% CI 6.0-10.7) for NHL.	Type of cancer (Melanoma Highest among those who developed CVD), Developed CVD (vs. not developed CVD)	No

Reference	Country of origin	Purpose	Data source	Cancer type	Diagnosis period	Age at diagnosis (years)	Survival entry date	Number of AYA participants	Follow-up time	Results	Statistically significant risk factors	Non-significant risk factors
Anderson C, 2019 [78]	United States	To examine risk of mortality from non-cancer causes after an AYA cancer diagnosis and investigate disparities according to race/ethnicity and other characteristics.	SEER	Any primary malignancy except Kaposi sarcoma	1987-2015	15-39	At diagnosis	401,287	Median 7.1 years for women and 6.1 years for men	The 10-year cumulative incidence of noncancer-related death after AYA cancer was 2% and 5% among women and men, respectively. With adjustment for cancer type, all noncancer mortality was increased among non-Hispanic Black AYAs (vs. non-Hispanic White: HRWomen = 2.31 (95% CI: 2.16–2.47); HRMen = 2.17 (95% CI: 2.05–2.30)) and those in the South (HR vs. Northeast: HRWomen = 1.18 (95% CI: 1.07–1.29); HRMen = 1.42(95% CI: 1.31–1.55)) or in rural counties (HR vs. metro: HRWomen = 1.74 (95% CI: 1.47–2.07); HRMen = 1.57 (95% CI: 1.33–1.86)). Mortality from CVD and infectious disease was also elevated among non-Hispanic Black AYAs. Compared to the lowest quintile of poverty, persons with the most poverty had a higher risk for noncancer death HR=1.4, 95% CI 1.3, 1.5).	All noncancer Deaths: non-Hispanic Black, Hispanic (vs Non-Hispanic White) for males and non-Hispanic Black (vs Non-Hispanic White) for females, living in West, South, and Midwest US (vs Northeast) for male, living in South US (vs Northeast) for females. living in a rural county (vs Metro) for males, living in a rural, urban area (vs Metro) for females, % of persons below poverty level quartiles Q2, Q3, Q4 (vs Q1) for both males and females, % Persons with < HS education quartiles Q3 & Q4 (vs Q1) for males and	All noncancer deaths: Other non-Hispanic (vs Non-Hispanic White) for males and Hispanic, Other non-Hispanic (vs Non-Hispanic White) for females, living in West, Midwest US (vs Northeast) for females, living in Urban setting (vs Metro) for males, % Persons with < HS education quartiles Q2 (vs Q1) for both males and females.

Reference	Country of origin	Purpose	Data source	Cancer type	Diagnosis period	Age at diagnosis (years)	Survival entry date	Number of AYA participants	Follow-up time	Results	Statistically significant risk factors	Non-significant risk factors
											females, type of cancer (Highest for NHL, leukemia, HL, head and neck, colorectal among males; and NHL, Leukemia and CNS among females).	
Bagnasco F, 2019 [79]	Italy	To evaluate overall and cause-specific mortality in a large cohort of Italian childhood cancer survivors and adolescent cancer survivors identified through the off-therapy registry.	Off-therapy registry of the Associazione Italiana di Ematologia ed Oncologia Pediatrica	1960-185: ALL, ANLL, Wilms tumor, HL, NHL, and neuroblastoma. 1986 to 1989 added Soft-tissue Sarcomas and CNS. All tumor types since 1989	1960-1999	15-21	5-years post-diagnosis	753	Median 22 years post-diagnosis (for censored individuals)		None presented	NA
Chao C, 2019 [25]	United States	To provide a comprehensive assessment of SMN risk in survivors of AYA cancer using study methods that minimized confounding to inform survivorship care planning for	SEER	Any primary malignancy	1990-2012	15-39	2 years post-diagnosis	10,574	Median 7.7 years	AYA cancer survivors with SMN were at 7-fold increased risk of dying compared with survivors who did not develop SMN (adjusted HR, 7.17, 95% CI, 6.06-8.49).	development of SMN (vs Not developing SMN)	No

Reference	Country of origin	Purpose	Data source	Cancer type	Diagnosis period	Age at diagnosis (years)	Survival entry date	Number of AYA participants	Follow-up time	Results	Statistically significant risk factors	Non-significant risk factors
		survivors of AYA cancer.										
Moke D, 2019 [80]	United States	To gain a more contemporary understanding of trends and disparities of site-specific AYA survival.	California Cancer Registry	Any primary malignancy	1988-2014	15-39	At diagnosis	225,493	NR	The 7 and 10-year overall survival probability was higher among those diagnosed in 2001-2017 compared to 1988-2000 (78.1% vs 66.7%) and (75.3%vs 64.4%), respectively.	Period of diagnosis (1988-2000 worse than 2001-2014)	No
Armenian S, 2020 [81]	United States	To examine cause-specific mortality in 2-year survivors of AYA cancers matched to individuals without cancer to determine whether mortality rates changed over time.	Kaiser Permanente Southern California and SEER	Any primary malignancy	1990-2012	15-39	2-years post-diagnosis	10,574	Median 10.1 years	Lowest long-term survival in breast cancer survivors (25 years: 59.8%) and the highest long-term survival in thyroid cancer survivors (25 years: 95.3%). Radiation therapy was associated with a higher risk of death from SMN (HR=1.5, 95% CI 1.0-2.1).	Overall Mortality: female sex (vs males) Younger age at diagnosis 15-19, Cancer type (Highest for Breast Cancer Survivors). Among cancer survivors SMS and Health-related deaths: Age at diagnosis 20-29, 30-39 (vs 15-19) for death from other health-related, female sex (vs male) for death from SMN, Asian/Pacific Islander (vs Non-Hispanic White-	Among Cancer Survivors: 20-29, 30-39 (vs 15-19) for death from SMN, Race/ethnicity Hispanic, Asian/Pacific Islander, Non-Hispanic Black (vs. Non-Hispanic White) for SMN deaths, Asian/Pacific Islander, Hispanic (vs Non-Hispanic White) for other health-related deaths

Reference	Country of origin	Purpose	Data source	Cancer type	Diagnosis period	Age at diagnosis (years)	Survival entry date	Number of AYA participants	Follow-up time	Results	Statistically significant risk factors	Non-significant risk factors
											Asian/pacific Islander at lower Risk) for death from SMN (HR=0.23, 95% CI 0.07-0.73) and non-Hispanic Black (vs Non-Hispanic White) for death from other health-related causes (HR=1.74, 95% CI 1.08-2.80), cancer stage at diagnosis not applicable, II, III/IV (vs Stage I) for both SMN and health-related deaths, radiation yes (vs No) for death from SMN (HR=1.45, 95% CI 1.03-2.06).	
Cuglievan B, 2020 [82]	United States	To determine association between lag time to diagnosis, health insurance type and income status with AYA cancer	The University of Texas MD Anderson Cancer Centre	Brain tumor, HL, Leukemia, non-HL, thyroid cancer, sarcomas	2001-2003	15-29	5-years post-diagnosis	201	NR	Late overall survival probability at 15 years post-diagnosis was higher for those with private insurance than those with public insurance (89%, 95%CI: 80%-94% vs 62%, 95%CI: 36%-80% respectively). The trend was observed among patients with brain tumors, leukemia, and	Public health insurance (vs. private); Household income (<=50,000). Poorest long-term overall	Age (15-19, 20-24, 25-29), Sex (male, Female), race/ethnicity (African American, Asian American,

Reference	Country of origin	Purpose	Data source	Cancer type	Diagnosis period	Age at diagnosis (years)	Survival entry date	Number of AYA participants	Follow-up time	Results	Statistically significant risk factors	Non-significant risk factors
		patients' long-term survival.		(bone or soft-tissue)						thyroid cancer. Significantly higher long overall survival also found for AYA residing in high-income neighborhoods that those in low-income neighborhoods.	survival for combination of public insurance and low income (<=50,000) at diagnosis	Hispanic, Non-Hispanic White)
Suh E, 2020 [42]	United States & Canada	To describe chronic health conditions and all-cause and specific mortality among survivors of early-adolescent and YA cancer.	Childhood Cancer Survivorship Study	Leukemia, CNS malignancy, HL, NHL, Wilms tumor, neuroblastoma, soft-tissue sarcoma, and bone cancer	1970-1999	15-20	5-years post-diagnosis	5,804	Median 20.6 years	SMR varied by cancer diagnosis, with the highest being for CNS (SMR=7.8, 95% CI 6.6-9.2) and the lowest being for NHL (SMR=3.8, 95% CI 3.1-4.7).	Type of cancer (HL and CNS Highest SMRs)	No
Lymphoma cohort												
Anton-Culver, 2010 [83]	United States	To examine whether socioeconomic factors other than race/ethnicity and treatment differences influence survival in AYA with NHL.	California Cancer Registry	NHL	1996-2005	15-39	At diagnosis	3,489	NR	The adjusted HR for all-cause mortality increased by 2% by year increase of age at diagnosis was 1.02 (1.01-1.03). Females had lower overall mortality than males (HR=0.65, 95% CI 0.57-0.75). The unadjusted overall survival at 100 months since diagnosis by neighborhood socio-economic status (SES) was approximately 73.7% among AYA living in Highest SES, 68.3% for High n SES, 67.8% for Middle SES, 57.5% for Low SES and 52.8% among those in the Lowest SES. Adjusted HR for all-cause mortality among people who did not have chemotherapy	Sex male, increasing age at diagnosis, stage (distant and regional worst compared to local); Nodality (extranodal worst compared to nodal), Not receiving chemotherapy as first line of treatment, neighborhood	

Reference	Country of origin	Purpose	Data source	Cancer type	Diagnosis period	Age at diagnosis (years)	Survival entry date	Number of AYA participants	Follow-up time	Results	Statistically significant risk factors	Non-significant risk factors
										was 1.27 (1.02-1.57) compared to people who did, while lymphoma-specific mortality was 0.37 (0.24-0.57).	socio-economic status (low vs. high)	
Castellino, 2011 [84]	United States	To identify factors affecting mortality risk in HL survivors.	Childhood Cancer Survivor Study	HL	1970-1986	15-21	5-years post-diagnosis	1,273	Median 23.8 and 16.1 years for participants alive and dead, respectively		None presented	NA
Xavier A, 2015 [31]	United States	To analyze SMN and survival outcomes of AYAs with early stage classical HL.	SEER	HL	1995-2010	15-39	6-months post-diagnosis	5,156	Median 97 months	5-year survival was better among patients treated with RT relative to those who were not (96.1% vs. 94.6%, respectively, p=0.002). In multivariable analysis, patients had a 34% higher risk of death if not treated with RT. Hispanics (HR=1.5, 95% CI 1.1-2.0) and non-Hispanic Blacks (HR=2.3, 95% CI 1.7-3.2) experienced an increased risk of death compared to non-Hispanic Whites.	Radiation therapy omission (vs. Use of RT), earlier era of diagnosis (1995-2002 vs. 2003-2010), male sex (vs. female), race/ethnicity (Hispanic, non-Hispanic Black vs. non-Hispanic White), histological subtype (Lymphocyte-depleted vs. Nodular sclerosis)	Histology (Classical NOS, Mixed cellularity, Lymphocyte-rich vs. Nodular sclerosis)
Hossain J, 2015 [85]	United States	To examine sex disparities in male	SEER	AML	1973-2012	15-24	At diagnosis	2,290	NR: Median survival time was 92 and 93	The risk of mortality was 30% greater for males compared to females in the 20-24 age group (HR 1.30, 95% CI 1.12-1.52). There was	Male sex (vs Female), for	Sex for patients 15-19 years of age

Reference	Country of origin	Purpose	Data source	Cancer type	Diagnosis period	Age at diagnosis (years)	Survival entry date	Number of AYA participants	Follow-up time	Results	Statistically significant risk factors	Non-significant risk factors
		versus female AML patients aged 0-24.							months for females and males, respectively	no statistically significant difference in survival in males vs. females in the 15-19 age group.	patients in their early 20s.	
Bhuller K, 2016 [32]	Canada	To highlight specific long-term morbidity and mortality issues for TYA patients with HL.	Childhood, Adolescent and Young Adult Cancer Survivors (CAYACS) Research Program in British Columbia; cases identified fro theBritish ColumbiaC ancer Registry	First primary malignancy : HL. SMN: Any secondary malignancy	1970-1999	15-24	5-years post-diagnosis	442	Median 19.6 years from diagnosis	During the 1970-1979 treatment era, SMR for all-cause mortality was 11.3 (95% CI 8.2-15.2; AER 13.4) compared to 4.9 (95% CI 2.5-8.8; AER 3.3) and 7.2 (95% CI 2.3-16.9; AER 3.9) for the 1980-1990 and 1990-1999 treatment eras. By far, the most unexpected deaths occured within 5-9 years of diagnosis (SMR 248.9, 95% CI 159.5-370.3; AER 191.1).	Treatment era (SMR for 1970-1979 higher than for 1980-1989), time since diagnosis (SMR for 5-9 years higher than all others 10+)	Sex (female vs. male), Chemotherapy (yes vs. no), Radiation therapy (yes vs. no), Surgery (yes vs. no)
Keegan T, 2016 [86]	United States	To evaluate the effect of socio-demographic characteristics, treatment and subsequent cancers on survival in AYAs diagnosed with HL.	California Cancer Registry	HL	1988-2011	15-39	At diagnosis	9,353	Mean 11 years	Among patients diagnosed with Stage I or II HL, the survival probability at 10- and 20-years were both 97% for radiation-therapy only, both 96% for "unknown" therapy, 95% and 93% for combined-modality therapy, and 93% and 91% for chemotherapy only. Among patients diagnosed with Stage III or IV HL, the survival probability at 10- and 20-years were 95% and 87% for radiation-therapy only, both 92% for "unknown" therapy, 89% and 88% for combined-modality therapy, and	Overall survival: Male (vs female), Age at diagnosis 35-39 (vs. 15-19), Early year of diagnosis 1982-1992 (vs. 2007-2011), Black and Hispanic race (vs. Non-Hispanic White), lower SES (lowest	Overall survival: Marital status at diagnosis (not married vs Married), Asian/Pacific Islander (vs. Non-Hispanic White), histologic subtype - Mixed cellularity, Lymphocyte

Reference	Country of origin	Purpose	Data source	Cancer type	Diagnosis period	Age at diagnosis (years)	Survival entry date	Number of AYA participants	Follow-up time	Results	Statistically significant risk factors	Non-significant risk factors
										86% and 84% for chemotherapy only. Compared to NH-Whites, overall survival was decreased among Blacks (HR=1.40, 95% CI 1.14-1.71) and Hispanics (HR=1.16, 95% CI=1.00-1.34), and HL-specific survival was decreased among Blacks (HR=1.62, 95% CI 1.24-2.11) and Hispanics (HR=1.35, 95% CI 1.12-1.64). Males experienced worst overall survivals than females (HR=1.3, 95% CI 1.2, 1.5).	quintile vs highest quintile), public or no insurance (vs Private/ military), No otherwise specified histologic subtype (vs Nodular sclerosis)	depletion, Lymphocyte rich (vs Nodular sclerosis), Urbanization level non-metropolitan, unknown (vs Metropolitan)
Keegan T, 2018 [67]	United States	To determine associations between sociodemographic factors and medical conditions among 2-year HL survivors.	California Cancer Registry and hospital data from the California Office of Statewide Health Planning and Development	HL	1996-2012	15-39	2-years post-diagnosis	5,085	Median 9.5 years	All medical conditions examined in this study reduced overall and HL-specific survival. Respiratory conditions reduced overall survival the most of any condition (HR 6.17, 95% CI 4.5, 8.5).	Medical condition Circulatory System Diseases (vs No), Respiratory system diseases (vs No), Chronic kidney disease/ renal failure (vs No), Liver disease (vs No) Endocrine and related diseases (vs No) Subsequent cancers (vs No)	Medical condition Hypothyroidism (vs No) for both overall and HL-specific survival; Subsequent cancers (vs No) for HL-Specific Survival
Patel C, 2018 [87]	United States	To determine late-effects of treatment in overall and HL-mortality in patients with early-stage HL	Multi-institutional database	HL	1968-2007	21-30	At diagnosis	511	Median 15.2 years		None presented	NA

Reference	Country of origin	Purpose	Data source	Cancer type	Diagnosis period	Age at diagnosis (years)	Survival entry date	Number of AYA participants	Follow-up time	Results	Statistically significant risk factors	Non-significant risk factors
		after radiation therapy.										
Leukemia cohort												
Goldman, 2010 [88]	United States, Australia, Brazil, Canada, England, France, Germany, Argentina, Austria, Belgium, Czechoslovakia, China, Denmark, Egypt, Finland, Hong Kong, Hungary, India, Iran, Ireland, Italy, Japan, Korea, Malaysia, Mexico, the Netherlands, New Zealand, Poland, Portugal,	To understand the risk of allogenic HCT transplantation in chronic myeloid leukemia in first chronic phase who survive in continuous remission for 5 years after transplantation, and to compare mortality rates with the general population.	Center for International Blood and Marrow Transplant Research	Chronic myeloid leukemia	1978-1998	20-39	5-years after HCT transplantation	1,373	Median follow-up 12 years from diagnosis and 11 years from HCT transplantation	The relative risk of death, treatment failure, or both among those 20-29 and 30-39 years of age at transplantation does not differ from those of patients age <20 years at HCT transplantation	None presented	NA

Reference	Country of origin	Purpose	Data source	Cancer type	Diagnosis period	Age at diagnosis (years)	Survival entry date	Number of AYA participants	Follow-up time	Results	Statistically significant risk factors	Non-significant risk factors
	Russia, Saudi Arabia, Scotland, South Africa, Spain, Sweden, Switzerland, Taiwan, Turkey, and Uruguay											
Chen Y, 2012 [89]	United States	To determine incidence and relative survival of acute promyelocytic leukemia (APL) during a 34-year period.	SEER	Acute promyelocytic leukemia	1975-2008	20-39	At diagnosis	372	NR	Ten-year relative survival (RS) was 0.24 (95%CI 0.16–0.33) for the 1975-1990 period, and 0.60 (95%CI 0.50–0.68) for the 1991-1999 period. Ten-year RS for the most recent period (2000-2008) was not reported.	None presented	NA
Hunger S, 2012 [90]	United States	To examine population-based improvements in survival and the impact of clinical covariates on outcome among children and adolescents with ALL (ALL) enrolled onto COG clinical trials between 1990 and 2005.	COG ALL clinical trials	ALL	Enrollment period: 1990-2005	15-22	At time of enrollment	1,515	Median 8.02 years	8% of adolescent ALL survivors in the cohort died between 5-9.99 years after the start of the study. <1 % died at 10+ years.	Enrollment era (lower survival probability for 1990-1994 compared to 1995-2005)	None stratified by age
Canner J, 2013 [91]	United States	To examine the outcomes of AYA patients aged 16-20	Children's Cancer	AML	1989-2006	16-20	At diagnosis	238	NR: At least 5 years	Overall survival for AYAs eight years after study entry was approximately 48%, compared to	None presented	NA

Reference	Country of origin	Purpose	Data source	Cancer type	Diagnosis period	Age at diagnosis (years)	Survival entry date	Number of AYA participants	Follow-up time	Results	Statistically significant risk factors	Non-significant risk factors
		relative to younger patients based on data from four consecutive COG trials.	Group and COG							approximately 58% for younger patients (< 16 years old).		
Woods W, 2014 [92]	United States	To determine if differences in pediatric versus adult oncology treatments affect outcomes among AYAs with AML.	COG, Cancer and Leukemia Group B an Southwest Oncology Group trials	AML	1986-2008	16-21	At diagnosis	517	NR	Ten-year overall survival was 45.6% and 34% among the COG and CALG/SWOG cohorts, respectively. Ten-year overall survival was higher for patients aged 16-18 compared to aged 19-21 (43% vs. 32%, p=0.034).	Age (older age at diagnosis 19-21 vs 18-18); Trial era (before 1996 worst than after 1996)	No
Wolfson J, 2018 [93]	United States	To evaluate the contribution of treatment site to outcome disparities experienced by AYA cancer survivors.	Los Angeles County Cancer Surveillanc e Program	ALL, AML	1998-2008	15-39	At diagnosis	761	NR	Seven year survival probability for 15-39 year old ALL survivors were approximately 38% and 56% for patients treated at non-CCC/COG (other) and CCC/COG (Comprehensive Cancer Centers/ COG) facilities, respectively. Seven year survival probability for 15-39 year old AML survivors were approximately 48% and 49% for patients treated at non-CCC/COG (other) and CCC/COG facilities, respectively.	tumor type, Treatment facility CCC/COG (vs Non-CCC/COG) for ALL patients, Older age at diagnosis(22-39) for ALL patients	Treatment facility CCC/COG (vs Non-CCC/COG) for AML
Zheng C, 2018 [94]	China	To analyze the outcomes of chronic myeloid leukemia in AYA patients receiving unrelated cord blood transplantation compared with those patients receiving sibling allogenic	Data from Anhui Provincial Hospital	Chronic myeloid leukemia	2002-2015	15-39	At diagnosis	74	Median 81 or 89 months depending on treatment	The seven-year overall and leukemia-specific survival for cord blood transplant patients were 55% and 48%, respectively, compared to sibling-allo-HCT which was 63% and 61%, respectively.	None presented	NA

Reference	Country of origin	Purpose	Data source	Cancer type	Diagnosis period	Age at diagnosis (years)	Survival entry date	Number of AYA participants	Follow-up time	Results	Statistically significant risk factors	Non-significant risk factors
		hematopoietic cell transplantation.										
Baron F, 2020 [95]	80 European centers	To evaluate the long-term impact of type of anthracycline given in the induction chemotherapy. Also, to evaluate the impact of hematopoietic stem cell transplantation(HLA identical sibling donor or an autologous) for patients who reach complete or incomplete remission.	EORTC/GIMEMA AML-10 trial	Primary or secondary AML	1993-1999	15-35	At diagnosis	661	Median 11 years	No difference in survival by randomization group type (MXR/IDA vs DNR) for AYA 15-25 (HR: 0.85, 95%CI: 0.56-1.27) or AYA 26-35 (HR: 1.11, 95%CI: 0.78-1.58). Similarly, no difference in survival was found by donor type (i.e., no donor vs donor) for AYA 15-25 (HR: 0.66, 95%CI: 0.4-1.1) or 26-35 (HR: 0.65, 95%CI: 0.4-1.03) or	None presented	NA
Venkitachalam R, 2020 [96]	United States	To examine temporal trends in overall survival and early mortality among pediatric acute promyelocytic leukemia.	SEER	Acute Promyelocytic Leukemia	1976-2016	16-20	At diagnosis	246	NR: Overall survival truncated at 7 years	The seven year survival probability was approximately 75.6% among AYA 16-20	None presented	NA
Melanoma cohort												
Fossa S, 2011 [97]	United States	To identify factors that increase risk of testicular cancer-specific mortality.	SEER	Testicular cancer	1978-2006	15-39	At diagnosis	20,411	97,127 person-years for 15-29 and 93849 for 30-39	Ten-year cumulative TC-specific mortality rate for seminoma and nonseminoma were 1.4% (95%CI 1.2% to 1.7%) and 6.1% (95%CI 5.7% to 6.7%), respectively. Significantly decreased in mortality was observed for participants aged 40 at diagnosis for seminoma HR:	Non-seminoma patients higher 10 year-cumulative mortality reported than in the seminoma	None stratified by age

Reference	Country of origin	Purpose	Data source	Cancer type	Diagnosis period	Age at diagnosis (years)	Survival entry date	Number of AYA participants	Follow-up time	Results	Statistically significant risk factors	Non-significant risk factors
										2.0 (95%CI 1.5 to 2.6); and nonseninoma HR: 2.1 (95% CI, 1.7 to 2.6)	group. No statistical test done for this. However we can see that the CI do not overlap between the two groups	
Pollack L, 2011 [98]	United States	To describe melanoma survival in the United States by demographics and cancer characteristics.	SEER	Melanoma excluding melanoma in situ	1992-2005	15-39	At diagnosis	13,383	NR: Based on exclusion criteria, minimum follow-up time is 5 years	10-year Melanoma-specific survival was 91.9%. AYA had better 10-year survival probability than those diagnosed at 40-64 (86.7%) or 65+(77.0%).	None presented	NA
Green A, 2012 [99]	Australia	To determine 20-year survival among people diagnosed with thin melanomas (<= 1.00 mm) in the general population.	Queensland Cancer Registry	Thin melanomas (<= 1.00 mm)	1982-2006	15-24	At diagnosis	1,381	NR	Ten, 15 and 20-year thin melanoma survival probabilities were 98.5%, 98.2% and 97.9% respectively. Better overall survival from thin melanomas for 15-24 years compared to those 45 or older at diagnosis.	None presented	NA
Reed K, 2012 [100]	United States	To determine the incidence of melanoma and overall disease-specific survival of young adults with cutaneous melanoma.	Rochester Epidemiology Project	Cutaneous melanoma	1970-2009	18-39	At diagnosis	256	Median 3.1 years for participants who died; Median 4.4 years for participants who were alive at last follow-up.	Ten-year overall survival probability by decade of diagnosis were approximately 82.7% for 1970-1979, 89.1% for 1980-1989, 94.3% for 1990-199, and 99.7% for 2000-2009.	Period of diagnosis (worst in earlier periods 1970-1979. Significant decrease in risk of death with each 1-year increased in calendar year of diagnosis)	Sex and histologic subtype

Reference	Country of origin	Purpose	Data source	Cancer type	Diagnosis period	Age at diagnosis (years)	Survival entry date	Number of AYA participants	Follow-up time	Results	Statistically significant risk factors	Non-significant risk factors
Gamba C, 2013 [101]	United States	To determine whether long-term survival varies between non-Hispanic White male and female AYA diagnosed with melanoma.	SEER	Invasive melanoma of the skin	1989-1999	15-39	At diagnosis	8,853	Mean follow-up 13.1 years for cases diagnosed 1989-1999	The results reported are for participants in the 1989-1999 diagnosis period. Overall, males are at high risk of mortality compared to females (HR: 1.45, 95% CI 1.25-1.67). Stratifying by age at diagnosis, the significantly increased risk in males was observed for males aged 30-34 (HR: 1.92, 95%CI 1.45-2.54) and 35-39 (HR: 1.28, 95%CI 1.03-1.60).	All comparisons made are for males vs. females: Male sex, age 30+ , histologic subtype (SSM, NM, NOS), tumor thickness (all significant except for ≥4.01), Presence of metastasis (cutaneous only, regional, unknown)	All comparisons are made for males vs females: Age at diagnosis (15-124, 25-29); tumor thickness(≥4.01); Distant metastasis
Plym A, 2014 [102]	Sweden	To compare clinical characteristics, management and survival between young adults and older adult melanoma patients in Central Sweden.	Regional Quality Register of Cutaneous Malignant Melanoma in Central Sweden (the Uppsala/Örebro Health Care Region)	Invasive malignant melanoma	1997-2011	15-39	At diagnosis	584	Median 4.9 years	Eight and 10-year cumulative relative survival were approximately 92.1% and 90.9% respectively	Stage of disease (Stage III worst)	No
Other tumor-specific cohort												
Smoll N, 2013 [103]	United States	To model the differences in survival of	SEER	Chordoma	1973-2009	16-39	At diagnosis	205	Median 4.7 years	Relative survival rates for AYAs were 69% (95% CI 60-76) , 59% (95%	None presented	NA

Reference	Country of origin	Purpose	Data source	Cancer type	Diagnosis period	Age at diagnosis (years)	Survival entry date	Number of AYA participants	Follow-up time	Results	Statistically significant risk factors	Non-significant risk factors
		chordoma patients across age groups in the face of differing expected mortality rates by using relative survival methods to assess the effect of age and sex on prognosis.								CI 49-68) and 56% (95% CI 44-66) at 10, 15 and 20 years, respectively.		
Youn P, 2014 [104]	United States	To quantify long-term, site-specific mortality risks associated with survival of adolescent bone and soft tissue sarcoma.	SEER	Bone and soft tissue sarcoma	1973-2007	15-39	At diagnosis	28,844	113,206 person-years	All-cause mortality in survivors was 76% higher compared to that of the general population (SMR 1.76, 95% CI 1.60-1.92; AER 19). At 20 years, this trend persisted (SMR 1.39, 95% CI 1.04-1.82; AER 20). Risk of mortality from reasons other than the original diagnosis were larger for patients with chemoresponsive vs. nonchemoresponsive sarcomas (SMR 2.76, 95% CI 2.20-3.41 vs. SMR 1.63, 95% CI 1.47-1.80). Chemotherapy (SMR 3.18, 95% CI 2.29-4.3), radiotherapy (SMR 4.54, 95% CI 1.8-4.67), or chemotherapy and radiotherapy (SMR 1.36, 95% CI 0.68-2.43) were associated with elevated mortality among patients with chemosensitive sarcoma. SMR was similarly elevated in treatment groups containing patients with nonchemosensitive sarcoma. Chemotherapy significantly contributed to excess deaths from second cancers (SMR=5.24, 95% CI 2.61-9.38) and noncancer causes (SMR=2.79, 95% CI 1.9-3.97).	Type of treatment (Significantly higher SMR for Chemo+/-surgery, radiation +/-surgery, and chemo+radiation+/-surgery), histology (Chemosensitive vs Nonchemosensitive)	Type of treatment: Surgery only for all causes of death (i.e., non-sarcoma, second cancer, non-cancer, and circulatory disease death) among chemosensitive sarcoma patients, and for second cancer and circulatory disease death among Nonchemosensitive sarcoma patients. Chemotherapy +/- Surgery for circulatory disease death among nonchemosensitive sarcoma.

Reference	Country of origin	Purpose	Data source	Cancer type	Diagnosis period	Age at diagnosis (years)	Survival entry date	Number of AYA participants	Follow-up time	Results	Statistically significant risk factors	Non-significant risk factors
												RT +/- Surgery for non-cancer death among Chemosensitive and circulatory disease death for both chemosensitive and nonchemosensitive sarcoma patients. Chemo and RT +/- Surgery for noncancer, and circulatory disease death among Chemosensitive sarcoma patients
Keegan T, 2015 [105]	United States	To evaluate survival among AYA men and by neighborhood SES, health insurance status, and detailed clinical factors to identify subgroups of young patients at higher risk of mortality from this cancer that is, otherwise, generally associated with excellent prognosis.	California Cancer Registry	First invasive thyroid carcinoma excluding Hürthle cell carcinomas	1998-2010	15-39	At diagnosis	16,827	Mean 10.3	Compared to women of the same age, AYA men were more likely to die from any cause after a diagnosis of thyroid cancer (HR 2.68,95% CI 2.14–3.34). Higher risk of death for AYAs diagnosed at 30-34 (HR:1.53, 95% CI 1.16–2.01) and 35-39 (HR: 2.01, 95% CI 1.54–2.62) years of age compared to those diagnosed at 15-29 survival than younger AYAs (HR 1.5-2.0)	Male sex (vs. Female), older age at diagnosis (30-34, 35-39 vs., 15-29), residing in lower SES neighbourhoods (low (quintile 1-2 vs. High (quintile 3-5)), residing in non-metropolitan areas (vs. Metropolitan),	Race/ethnicity(African American, Hispanic, Asian/Pacific vs. Non-Hispanic White); Histology (Follicular vs. Papillary); Lymph node dissection (yes, unknown vs. No); Total thyroidectomy

Reference	Country of origin	Purpose	Data source	Cancer type	Diagnosis period	Age at diagnosis (years)	Survival entry date	Number of AYA participants	Follow-up time	Results	Statistically significant risk factors	Non-significant risk factors
											being unmarried (vs. Married), having subsequent cancer (vs. no). Public/no insurance (vs. Private/military insurance).	(yes vs. No); Radioactive Iodine (yes vs. No); Hormone therapy (yes vs. no)
DeRouen M, 2016 [106]	United States	To study the independent association between race/ethnicity and/or neighborhood SES and survival in AYAs with testicular cancer.	SEER	Testicular cancer	1988-2010	15-39	At diagnosis	14,249	Average 11.3 years for censored participants.	Higher risk of overall mortality observed for: Black (HR: 1.41, 95%CI 1.01–1.97) and Hispanic AYA (HR: 1.21 95%CI 107–1.37) compared to White; AYA from middle (HR: 1.34, 95% CI 1.12–1.60) and low (HR: 1.79, 95%CI 1.50–2.14) SES neighborhoods compared to those from high SES; Not married compared to married (HR: 1.57, 95% CI 1.39–1.78). Lower risk of mortality observed in AYA diagnosed at 15-24 compared to AYA 25-39 (HR: 0.70, 95%CI 0.61–0.80), and for those in more recent diagnostic eras (1996-2003 and 2004-2010) compared to 1998-1995 (HR: 0.77, 95% CI 0.67-0.88 and HR: 0.75, 95C% CI 0.64-0.87, respectively).	Neighborhood SES (middle and low worst survival than high SES), race/ethnicity (Hispanic and Black worse than White for nonseminoma), nonseminoma cancer, longer time to treatment (for those with nonseminoma), older age at diagnosis (25-39 for nonseminoma), marital status (unmarried AYA worse than married), period of diagnosis (1988-1995 worst	Race/ethnicity (Back, Asian/PI, Hispanic, Other vs. White for seminoma)

Reference	Country of origin	Purpose	Data source	Cancer type	Diagnosis period	Age at diagnosis (years)	Survival entry date	Number of AYA participants	Follow-up time	Results	Statistically significant risk factors	Non-significant risk factors
											compared to 2004-2010 for seminoma).	
Lau B, 2016 [107]	United States	To investigate whether age at primary malignancy is an independent predictor of overall survival (OS) for AYA patients diagnosed with secondary thyroid cancer who have survived ≥5 years from their primary malignancy.	SEER-9 & SEER-13	Thyroid SMN after 5 years post-diagnosis of any primary non-thyroid malignancy	1973-2010	15-39	5-years post-diagnosis	357	NR: Median overall survival was 30+ years	Compared to those diagnosed with a first primary at 0-14 years of age, AYA diagnosed with first primary at age 15-39 had significant lower OS at 10 (AYA: 83.6% vs. Pediatric:96.4%), 20 (56.3% vs. 88.0%) and 30 (50.9% vs.88.0%) years post-diagnosis.	Older age at diagnosis (vs 0-14), Black race (vs White), male gender (vs Female), having more than two tumors, more extensive primary disease	None stratified by age mentioned in the text or tables
Novetsky Friedman D, 2017 [108]	United States	To describe early and late overall mortality, cause-specific mortality, and key adverse health outcomes in a large, single-institutional cohort of patients with Ewing sarcoma.	Memorial Sloan Kettering, USA	Ewing sarcoma	1974-2012	20-39	At diagnosis	97	Median 7.8 years	HR for all-cause mortality was 3.0 (95% CI 1.4–6.4) for 20-29 year olds and 4.5 (95% CI, 2.0–10.6) for 30-39 year olds, compared to 0-9 year olds.	older age at diagnosis 20-29, 30-39 (vs 0-9)	None stratified by age at diagnosis
Bownes L, 2018 [109]	United States	To examine if SES impacts survival in AYAs with malignant ovarian germ cell tumors.	The National Cancer Data Base (NCDB)	Malignant ovarian germ cell tumors	1998-2012	15-39	At diagnosis	3,125	NR	Decreased survival was observed for those from a lower income quartile without insurance and with lower education background. The adjusted cumulative survival at 100 months from diagnosis by education measured as percentage with no high school were approximately 97.9%, 95.2% , 96.1%, and 95.6% among those with >=21%, 13.0 to 20.9%, 7.0-	Level of education (>= 21% without high school degree vs. <7%)	Insurance type (Government, Private vs. None), Median income quartile ($63,000 vs. <$38,000),

Reference	Country of origin	Purpose	Data source	Cancer type	Diagnosis period	Age at diagnosis (years)	Survival entry date	Number of AYA participants	Follow-up time	Results	Statistically significant risk factors	Non-significant risk factors
										12.9% and <7.0% without high school degree, respectively (P=0.017). The adjusted 100-months post diagnosis cumulative survival by median income quartiles 2006-2012 were approximately 97.4%, 95.0% , 97.1%, and 95.3% among those with median income <$38,000, $38,00-$47,999, $48,000-$62,999, and $63,000+, respectively (p=0.022). The adjusted 100-months post-diagnosis cumulative survival by insurance type was approximately 92.7%, 92.7% , and 94.4% among those without, government and private insurance, respectively (p=0.019).		
Challapalli S, 2018 [110]	United States	To describe nonclinical factors associated with head and neck survivorship among AYAs.	SEER-18	Head and neck squamous cell carcinoma	2007-2014	15-39	At diagnosis	1,777	NR: Analysis based on 8 years of follow-up	Survival rate after 8 years of follow-up: 73%. Similar survival differences by age at diagnosis. Increased hazard of death from head and neck cancer, specifically, for AYA on Medicaid (adjusted HR = 1.61, 95% CI 1.22–2.12) or uninsured (HR = 1.51, 95% CI 1.03–2.21) compared with those with private insurance; for people who did not receive surgery vs. those who did (HR=1.58, 95% CI 1.17-2.13).	uninsured/ Medicaid (vs Private), non-Hispanic other (vs. non-Hispanic White), higher stage of cancer Iv, II, II, Unstaged/unknown (vs stage I), type of head and neck cancer (oral cavity was worst-Referent Vs Hypopharynx/larynx, Nasopharynx, Oropharynx),	Age at diagnosis (15-29, 30-34 and 35-39); Year of diagnosis; Male sex (vs Female); Race/ ethnicity Hispanic, non-Hispanic Black (vs non-Hispanic White); Overall race/ethnicity variable not significant; site sinonasal (vs Oral cavity), Radiation No/unknown

Reference	Country of origin	Purpose	Data source	Cancer type	Diagnosis period	Age at diagnosis (years)	Survival entry date	Number of AYA participants	Follow-up time	Results	Statistically significant risk factors	Non-significant risk factors
											did not receive surgery (vs received surgery),	(vs Received radiation)
Chen I, 2018 [111]	United States	To compare AYA and pediatric overall survival in the most common pediatric solid tumors.	SEER	Extracranial solid tumors: Ewing sarcoma, neuroblastoma, osteosarcoma, rhabdomyosarcoma, and Wilms tumors.	1973-2010	15-39	At diagnosis	4,128	NR	Ten- and 20-year overall survival for AYA patients were, respectively, 42% and 38% for Ewing sarcoma, 30% and 29% for neuroblastoma, 56% and 53% for osteosarcoma, 41% and 39% for rhabdomyosarcoma, and 59% and 57% for Wilms tumor. Compared to pediatric age group (0-15) AYA are at higher risk of dying from all of the cancers studied except for osteosarcoma	tumor type (from survival curve, Neuroblastoma seems to be worst survival than Ewing sarcoma, osteosarcoma, rhabdomyosarcoma, and Wilms tumor)	No
Chu Q, 2020 [112]	United States	To evaluate factors associated with worse survival among breast cancer survivors based on urban versus rural residency.	Louisiana tumor Registry	Breast Cancer-women stage I to III	2004-2016	18-39	At diagnosis	1,492	Median 74 months	Taken AYA 18-39 years of age as referent category, the overall survival was similar than those 40-49 (HR:1.01 , 95%CI:0.87- 1.17) and 50-59 (HR:1.147, 95%CI: 0.99-1.32). However, it those diagnosed at ages 60-69 and 70+ had higher risk of mortality than AYA 18-39 (HR:1.68, 95%CI: 1.46- 1.94) vs HR:3.93, 95%CI: 3.40- 4.53) respectively. For cancer-specific survival, the only difference in risk of survival was found on those diagnosed at age 70 or more compared to AYA 18-39.	None presented	NA
Perisa M, 2020 [69]	United States	To examine clinical features at presentation, treatment factors, toxicities, and	Nationwide Children's Hospital	Ewing Sarcoma	1990-2017	15-39	At diagnosis	45	Median 98 months	Higher risk of mortality in AYA compared to pediatric patients (HR: 3.10, 95%CI:1.45-6.63).Ten year overall survival was approximately	None presented	NA

Reference	Country of origin	Purpose	Data source	Cancer type	Diagnosis period	Age at diagnosis (years)	Survival entry date	Number of AYA participants	Follow-up time	Results	Statistically significant risk factors	Non-significant risk factors
		outcomes in Ewing sarcoma AYA survivors compared with pediatric patients.	Columbus, Ohio							45% for AYA and 64% for pediatric patients		

Abbreviations: AER, absolute excess risk; ALL, acute lymphoblastic leukemia; AML, acute myelogenous leukemia; AYA, adolescent and young adult; CI, confidence interval; CNS, central nervous system; CVD, cardiovascular disease; HL, Hodgkin lymphoma; HR, hazard ratio; IRR, incidence rate ratio; NHL, non-Hodgkin lymphoma; NR, not-reported; RR, rate ratio; SES, socioeconomic status; SBDR, standardized bed day ratio; SHR, standardized hospitalization ratio; SIR, standardized incidence ratio; SMN, second malignant neoplasm; SPN, second primary neoplasm; YA, young adult.
